# Mechanistic study on the alleviation of postmenopausal osteoporosis by *Lactobacillus acidophilus* through butyrate-mediated inhibition of osteoclast activity

**DOI:** 10.1038/s41598-024-57122-x

**Published:** 2024-03-25

**Authors:** Junjie Dong, Guizhao Shu, Jin Yang, Bing Wang, Lingqiang Chen, Zhiqiang Gong, Xiaofeng Zhang

**Affiliations:** The First Affiliated Hospital of Kunming Medical University, Kunming, China

**Keywords:** Postmenopausal osteoporosis, *Lactobacillus acidophilus*, Butyrate, Osteoclasts, B cells, Intestinal bone axis, Cell biology, Immunology, Microbiology, Biomarkers

## Abstract

In China, traditional medications for osteoporosis have significant side effects, low compliance, and high costs, making it urgent to explore new treatment options. Probiotics have demonstrated superiority in the treatment of various chronic diseases, and the reduction of bone mass in postmenopausal osteoporosis (PMOP) is closely related to the degradation and metabolism of intestinal probiotics. It is crucial to explore the role and molecular mechanisms of probiotics in alleviating PMOP through their metabolites, as well as their therapeutic effects. We aim to identify key probiotics and their metabolites that affect bone loss in PMOP through 16srDNA sequencing combined with non-targeted metabolomics sequencing, and explore the impact and possible mechanisms of key probiotics and their metabolites on the progression of PMOP in the context of osteoporosis caused by estrogen deficiency. The sequencing results showed a significant decrease in *Lactobacillus acidophilus* and butyrate in PMOP patients. In vivo experiments confirmed that the intervention of *L. acidophilus* and butyrate significantly inhibited osteoclast formation and bone resorption activity, improved intestinal barrier permeability, suppressed B cells, and the production of RANKL on B cells, effectively reduced systemic bone loss induced by oophorectomy, with butyric acid levels regulated by *L. acidophilus*. Consistently, in vitro experiments have confirmed that butyrate can directly inhibit the formation of osteoclasts and bone resorption activity. The above research results indicate that there are various pathways through which *L. acidophilus* inhibits osteoclast formation and bone resorption activity through butyrate. Intervention with *L. acidophilus* may be a safe and promising treatment strategy for osteoclast related bone diseases, such as PMOP.

## Introduction

Postmenopausal Osteoporosis (PMOP) is a condition marked by disrupted bone metabolism, where bone loss exceeds bone gain due to the deficiency of estrogen following menopause^1,2^. It is a common chronic condition among elderly women and a prevalent cause of various fractures, significantly impacting the quality of life in elderly women and increasing morbidity and mortality rates. Literature has reported that bone loss in PMOP patients is a more important factor in the development of osteoporosis^3^. Therefore, inhibiting the formation of osteoclasts may be a crucial factor in improving PMOP symptoms.

In recent times, the gut-bone axis has emerged as a novel approach for preventing and treating PMOP. The gut-bone axis refers to the interaction between the microbiota in the gut and bone cells, which can alter bone metabolism through various mechanisms, including the production of metabolites, influencing host metabolism, and modulating the immune system^4^. Gut microbiota refers to a collection of microorganisms that inhabit the host’s intestines and have a significant impact on the host’s health^5^. Studies have reported that regulating gut microbiota can be a safer method to effectively improve PMOP^6–8^. Among these approaches, the role of probiotics cannot be overlooked, which are defined as “live microorganisms that, when administered in sufficient amounts, provide a benefit to the host’s health”^9^. The effect of probiotics on the intestinal microbiota is very prominent in the treatment of human and animal diseases. Its huge potential has prompted researchers to continue to deepen their research on probiotics^10^.Lactobacillus and Bifidobacterium are common probiotics found in everyday life, they can affect bone metabolism by changing the intestinal microbiota^11,12^.

Research indicates that probiotics can influence bone metabolism through certain pathways, thereby improving PMOP. A study by Jia et al. showed that probiotics can enhance PMOP by regulating gut microbiota and intestinal barrier function^13^. Britton et al. also reported that Lactobacillus reuteri could improve PMOP by inhibiting osteoclast formation^14^. Therefore, probiotics may serve as a biological marker for the treatment of PMOP, although their underlying mechanisms require further investigation.

Currently, research on the impact of probiotic metabolites on substance metabolism has become mainstream. Short-chain fatty acids (SCFAs) mainly include acetic acid, propionic acid and butyric acid, etc.^15^, as a critical metabolic product of gut probiotics, have been widely mentioned in many studies^16^. Studies have shown that SCFAs as a important metabolites of intestinal microorganisms, can participate in the regulation of bone metabolism by affecting local or immune functions^17^. Among them, propionic acid and butyric acid play an important role^17^. Research by Sonakshi Rastogi and colleagues suggests that Bifidobacterium breve can influence bone metabolism through the production of SCFAs^11^. Wu et al. mentioned in their report that Bifidobacterium bifidum can affect bone metabolism through its metabolite SCFAs^18^. Combining the above reports, it is evident that SCFAs may be an important factor in how gut probiotics influence bone metabolism. According to the relevant literature, SCFAs can also regulate immune cells and have biological effects^19^. Research by Yang W et al. indicates that SCFAs can help maintain intestinal homeostasis by modulating T cells^6^, and Zaiss MM et al. have mentioned in their reports that SCFAs can mediate Tregs and CD8+ T cells to impact bone metabolism^20^. However, there is limited literature on the effects of SCFAs on B cells. Sapra et al. mentioned in their report that RANKL produced by B cells contributes to the increase in osteoclasts^21^. Therefore, exploring whether SCFAs can inhibit the production of RANKL on B cells and affect bone metabolism is particularly important. Based on the literature mentioned above, intervening in the regulation of probiotics in the gut to modulate their metabolic products mediated by the immune system may be an effective approach to treat PMOP.

Based on the above literature reports, this article hypothesizes that a certain dominant bacterial group in probiotics can inhibit the formation of osteoclasts through its influence on short-chain fatty acids, thereby improving postmenopausal osteoporosis.

In order to explore novel and effective probiotics and their metabolites for improving PMOP, this study will employ 16S rRNA sequencing in combination with untargeted metabolomics to explore the primary gut probiotics and their metabolites associated with postmenopausal osteoporosis. The study will utilize an ovariectomized (OVX) mouse model as an experimental subjects to investigate their impact on bone metabolism and potential mechanisms.

## Methods

### Experiment statement

With the approval of the First Affiliated Hospital of Kunming Medical University Ethics Committee, we collected serum and fecal samples from both a normal group and a postmenopausal osteoporosis (PMOP) group.

We confirm all methods were carried out in accordance with relevant guidelines and regulations.

We are confirming that informed consent was obtained from all subjects and/or their legal guardian.

### Clinical sample collection

With the approval of the First Affiliated Hospital of Kunming Medical University Ethics Committee, we collected serum and fecal samples from both a normal group and a postmenopausal osteoporosis (PMOP) group. We obtained 10 replicate samples from each group for metabolomics and 16S rDNA amplicon sequencing to investigate specific microorganisms and their metabolites that may contribute to the onset of the condition.

Inclusion Criteria: Females aged 50–70 years who have been postmenopausal for more than 1 year. Samples were selected from patients undergoing medical examinations. Ten women with bone density T-scores ≥ − 1 SD were included in the normal bone mass group, while 15 women with bone density T-scores ≤ − 2.5 SD were included in the PMOP group.

Exclusion Criteria: Patients with secondary osteoporosis caused by conditions such as primary hyperparathyroidism, hyperthyroidism, type 1 diabetes, rheumatoid arthritis, and other secondary causes. Individuals with secondary osteoporosis due to long-term use of glucocorticoids. Patients with severe cardiovascular or cerebrovascular diseases, malignant tumors, or who had undergone menopause before the age of 45 or had undergone ovarian removal before the age of 50 without estrogen replacement therapy were also excluded.

#### Sample preprocessing and metabolite extraction

Serum samples stored at − 20 °C, along with quality control (QC) samples, were thawed in a 4 °C refrigerator until no visible ice remained inside the samples. Each sample (including QC) was mixed with 100 µL and the remaining samples were maintained in a frozen state. Then, 700 µL of extraction solution containing internal standard 1 (methanol: acetonitrile: water = 4:2:1, V/V/V) was introduced into each sample and QC. The mixture was shaken for 1 min and placed in a − 20 °C freezer for 2 h. Afterward, the samples were subjected to centrifugation at 25,000 g and 4 °C for 15 min. 600 µL of supernatant was carefully transferred to new EP tubes, and the samples were dried using a vacuum concentrator. Then, 180 µL of methanol: water (1:1, v/v) was introduced to dissolve the dried samples, and the mixture was vortexed for 10 min until it was fully dissolved in the reconstitution solution. Following this, the samples were subjected to centrifugation at 25,000 g and 4 °C for 15 min, and the resulting supernatant was transferred to new EP tubes. For quality control purposes, 20 μL from each sample was combined to create a QC sample. The prepared supernatants were then subjected to UPLC-MS analysis. Metabolite separation and detection were performed utilizing a Waters UPLC I-Class Plus system (Waters, USA) coupled with a Q Exactive high-resolution mass spectrometer (Thermo Fisher Scientific, USA). The spray voltages were configured at 3.8 kV for the positive ion mode (ESI+) and − 3.2 kV for the negative ion mode (ESI−).

#### Data preprocessing and quality control

The outcomes generated by Compound Discoverer were brought into metaX for initial data processing. The data preprocessing includes: normalization of the data utilizing Probabilistic Quotient Normalization (PQN) to obtain the relative peak areas; correction of batch effects utilizing QC-RLSC (Quality control-based robust LOESS signal correction); correction of the relative peak areas of all QC samples using QC-RLSC (Local Polynomial Regression Fitting Signal Correction); and correction of the relative peak areas of all QC samples. QC-RLSC (Quality control-based robust LOESS signal correction) was used to eliminate batch effect, and any compounds exhibiting a Coefficient of Variation (CV) in relative peak area exceeding 30% were excluded from all QC samples.

#### Differential metabolite selection

Differential metabolites between the two biological groups were selected using both univariate and multivariate analyses. Initially, overall differences between the two groups were examined using Principal Component Analysis (PCA) and Partial Least Squares-Discriminant Analysis (PLSDA). Subsequently, for the selection of differential metabolites, Variable Importance in Projection (VIP) values from Orthogonal Partial Least Squares Discriminant Analysis (OPLSDA) were used, with a fallback to PLSDA VIP values if overfitting occurred. Additionally, univariate analysis was employed, considering Fold Change and p-values. A volcano plot was generated to visualize the results. Differential metabolites were selected based on the following criteria: (1) VIP values from the OPLS-DA model ≥ 1; (2) Fold Change ≥ 1.2 or ≤ 0.83; (3) *P*-value < 0.05.

#### 16S sequencing data filtering

The PCR reaction system was prepared using 30 ng of high-quality genomic DNA samples along with the appropriate fusion primers, and the reaction parameters were set to perform PCR amplification, and the PCR amplification products were subjected to purification using Agencourt AMPure XP magnetic beads, dissolved in Elution Buffer, labeled, and then completed the library construction. The fragment range and concentration of the libraries were analyzed by Agilent 2100 Bioanalyzer. After evaluation, the libraries that met the quality criteria were chosen for sequencing on the HiSeq platform, considering their insert size.

The original sequencing data underwent the following processing steps to obtain Clean Data: Low-quality bases were removed using a sliding window approach. Specifically, a 25 bp window was used, and if the average quality score in the window was below 20, bases from the start of the window were trimmed. Sequences were discarded if the length after trimming was less than 75% of the original read length. Reads containing adapter contamination were removed, with a default adapter sequence overlap of 15 bp allowed and up to 3 mismatches. Reads containing ‘N’ characters were removed. Low-complexity reads were removed, with reads containing a continuous stretch of 10 or more identical bases considered low-complexity. Samples were differentiated based on barcodes, and barcode sequences were aligned to the sequencing reads with no allowed mismatches (0 bp).

#### Tags concatenation

Sequence concatenation was performed using the software FLASH (Fast Length Adjustment of Short reads, v1.2.11). It involved assembling paired-end reads obtained from double-ended sequencing into a single sequence, resulting in Tags from the high-variable regions. The concatenation conditions were as follows: a minimum matching length of 15 bp and an allowed mismatch rate of 0.1 within the overlap region. The software used for this process was FLASH (Fast Length Adjustment of Short reads, v1.2.11).

#### OTU clustering results and statistics

There are two methods for OTU clustering.Usearch: generates OTUs by clustering according to 97% sequence similarity; DADA2: generates ASV sequences by clustering denoised sequences with 100% similarity, collectively referred to here as OTUs.

Usearch:The spliced Tags were grouped into OTUs through the utilization of the software USEARCH (v7.0.1090).

DADA2: The DADA2 (Divisive Amplicon Denoising Algorithm) method within the QIIME2 software was utilized for denoising to obtain Amplicon Sequence Variants (ASVs), where ASVs are 100% identical sequences. Subsequently, a feature table (Feature, a collective term for ASVs/ASVs, etc.) is generated.

#### Integrated metabolome-16S analysis

We conducted Spearman correlation analysis between the significantly different secondary metabolites identified through metabolomics and the significantly different taxonomic groups obtained from 16S sequencing analysis. This allowed us to determine relationships between different taxonomic groups, different metabolites, and between different metabolites and taxonomic groups. Based on the calculated results, suitable filtering criteria were applied to obtain the final correlation relationships and network diagrams between different metabolites. In the results, “R” represents the correlation coefficient between different metabolites and different taxonomic groups, and a positive correlation coefficient is marked with a “+” sign, while a negative one is marked with a “−” sign.

### OVX model establishment

11 week old SPF grade female C57BL/6 J mice were randomly divided into Sham group (sham surgery group) and OVX group. Bilateral oophorectomy was performed on OVX group and OVX + metabolite Y group (low, medium, and high doses) mice under anesthesia to induce osteoporosis. In the sham surgery group, ovaries were only externalized but not removed. Mice were taken and placed in a gas anesthesia box with 3.5% isoflurane to induce anesthesia for 60–90 s; 1.5% isoflurane was used to maintain anesthesia. The mice were positioned in a supine posture, and the hair near the abdomen was removed and sterilized; after the mice were injected with anesthetics and entered into deep anesthesia for 3–5 min, the fallopian tubes and ovaries were separated and exposed, and the tubes were ligated at the proximal end of the ovary; the ovary and part of the fallopian tube were removed along the site of the ligation; the muscularis mucrosa and the skin were tightly sutured to complete the resection of the other side of the ovary and the sutures were closed. Paracetamol was used for pain relief for 24 h postoperatively. In the OVX + La-02 group, La-02 intervention was started in the 10th week after modeling (Gastric lavage intervention), with a La-02 concentration of 10^8^ CFU/mL and 0.2 mL/d/10 g for 2 weeks by continuous gavage; in the OVX + C4 group, C4 was supplemented in the drinking water after 4 weeks of modeling, with a final concentration of 75 mM, 150 mM, and 300 mM changed every 3 days for 8 weeks, (OVX for drinking water administration) .

### Micro-CT analysis of skeletal structural features in mice left femur

The left femur was taken and fixed with paraformaldehyde overnight, and Micro-CT scanning (Nikon, XT H 225/320LC) was performed and analyzed to characterize the skeletal structure of mice. The scanning voltage was 50 kVp, the current was 200 μA, and the integration time was 400 ms. The images were reconstructed and analyzed using VGStudio MAX 2.2 (VolumeGraphics, Heidelberg, Germany), and the software automatically calculated the following 3D indices, bone mineral density (BMD), bone volume divided by tissue volume (BV/TV), trabecular number (Tb.N), trabecular thickness (Tb.Th), ratio of bone surface area to bone volume (BS/BV), and ratio of bone surface area to tissue volume (BS/TV).

### Hematoxylin and eosin (HE) staining

The right femurs from each group of mice were fixed overnight in 4% paraformaldehyde at 4 °C. After decalcification in 10% EDTA for 21 days, the samples underwent ethanol gradient dehydration (2 days in 70% ethanol, 2 days in 96% ethanol, 2 days in 100% ethanol) followed by clearing in acetone for 8 h. Subsequently, the samples were embedded in paraffin wax. Thin sections with a thickness of 5 μm were obtained from the embedded samples, and these sections were subjected to staining with hematoxylin and eosin (HE).

### Immunohistochemistry (IHC) experiment

To evaluate intestinal permeability, immunohistochemistry staining was performed on 5 μm-thick sections obtained from the previously mentioned HE-stained samples. The sections were subjected to antigen retrieval using 0.01 M citrate buffer, followed by blocking with 3% H2O2 at room temperature for 20 min. Subsequently, they were incubated with 5% bovine serum albumin (BSA) in phosphate-buffered saline (PBS) at 37 °C for 30 min to block non-specific binding. Primary antibodies against MMP9 (abcam, ab760003), NFATc1 (invitrogen, PA5-79730), claudin 2 (invitrogen, 32-5600), claudin 3 (abcam, ab15102), and claudin 15 (invitrogen, 38-9200) were applied to the sections and allowed to incubate overnight. Afterward, secondary antibodies were incubated at 37 °C for 30 min. The sections were then subjected to DAB staining followed by counterstaining with hematoxylin. After dehydration, clearing, and mounting, the sections were analyzed.

To assess osteoclastogenesis, we used a tartrate-resistant acid phosphatase (TRAP) staining kit (Beyotime, P0332) to perform acid phosphatase activity staining.

### C4 content detection

For the C4 content detection, all reagents, samples, and standards were prepared according to the ELISA Kit for Butyric Acid (Cloud-Clone Corp, CEO777Ge). Each well was loaded with 50 µL of either the standard or the sample. Then, 50 µL of prepared assay reagent A was immediately added to each well, followed by shaking and mixing. The plate was incubated at 37 °C for 1 h. After incubation, the wells were aspirated and washed three times. Subsequently, 100 µL of prepared assay reagent was added to each well, and the plate was incubated at 37 °C for 30 min. Following this incubation, the wells were aspirated and washed five times. Next, 90 µL of substrate solution was added to each well, and the plate was incubated at 37 °C for 10–20 min. Finally, 50 µL of the stop solution was added, and the absorbance was immediately read at 450 nm using an ELx800 plate reader (BioTek, USA).

### Flow cytometry experiment

Fresh bone tissue was washed with PBS, and then marrow was extracted from the bone using a syringe. The marrow was incubated at 4 °C for 20 min with red blood cell lysis buffer (Elabscience, E-CK-A106). After incubation, the cells were washed twice with PBS to obtain bone marrow cells. Each sample tube contained 1 × 10^5^ cells, and 100 μL of Buffer was added to each tube. CD5 (invitrogen, MA5-17407), CD19 (invitrogen, MA5-32544), CD45R (B220) (invitrogen, 11–0460-82), and RANKL (invitrogen, MA5-16156) were added to the tubes, mixed well, and incubated at room temperature in the dark for 20 min. Stained samples were collected using a BD FACSCalibur flow cytometer (BD, FACS101), and 10,000 cells were collected for each sample. Each sample was analyzed in triplicate.

### RAW264.7 cell culture

RAW264.7 cells in the logarithmic growth phase were harvested and counted. They were then seeded into 12-well plates at a density of 1 × 10^5^ cells per well. After the cells adhered to the wells, the medium was replaced, and RANKL (MCE, HY-P7425) was added at a concentration of 50 ng/mL to stimulate the cells. The medium was changed every 2 days, and additional RANKL was supplemented to induce the formation of multinucleated osteoclasts. At the time points of 3 days, 7 days, and 14 days of osteoclast induction, the culture medium was supplemented with La-02 supernatant at a 1:20 ratio with the regular culture medium, and butyric acid sodium was added at a concentration of 1 mM for intervention.

### RT-PCR for detection of osteoclastogenesis-related gene expression

Total RNA was extracted from cells in each group, followed by reverse transcription reactions. Subsequently, qPCR reactions were carried out using the 2 × Universal Blue SYBR Green qPCR Master Mix kit. The qPCR program consisted of the following steps: 95 °C pre-denaturation for 1 min; 40 cycles of denaturation at 95 °C for 20 s, annealing at 55 °C for 20 s, and extension at 72 °C for 30 s. Table 1 presents a compilation of the primer sequences.

**Table 1:** Primers and sequences.

Gene	Forward (5′-3′)	Reverse (5′-3′)
GAPDH	CCTTCCGTGTTCCTACCCC	GCCCAAGATGCCCTTCAGT
CTSK	AATACCTCCCTCTCGATCCTACA	TGGTTCTTGACTGGAGTAACGTA
Acp5	CACTCCCACCCTGAGATTTGT	CATCGTCTGCACGGTTCTG
c-Fos	CGGGTTTCAACGCCGACTA	TTGGCACTAGAGACGGACAGA

### Immunofluorescence (IF) experiment

Cells were fixed in a 4% PFA solution at room temperature for 30 min. Afterward, they were blocked in PBS containing 2% Triton X-100 (PBST) and 5% goat serum for 1 h. Subsequently, the cells were incubated with the following primary antibodies overnight at 4 °C: F-actin (abcam, ab184675), MMP9 (abcam, ab76003), and NFATc1 (invitrogen, PA5-79,730). Following primary antibody incubation, the cells were incubated with secondary antibodies at 37 °C for 1 h. Imaging and analysis of the cells were performed using a fluorescence microscope (Carl Zeiss, Dragonfly 600).

### Osteoclast resorption activity assay

The experiment was conducted using the Bone Resorption Assay Kit (Cosmo Bio Co., LTD, CSR-BRA-48KIT). RAW264.7 cells from each intervention group were seeded at a density of 1 × 10^4^ cells/mL in FACS-labeled CaP plates containing DMEM culture medium with 10% fetal bovine serum. Then, 0.5 mL of osteoclast differentiation inducer RANKL was added, and the cells were cultured for 5–6 days. Afterward, 100 μL of conditioned culture medium from each group was transferred to a 96-well plate. Then, 50 μL of bone resorption assay buffer was added to each well, mixed, and the fluorescence intensity was measured with an excitation wavelength of 485 nm and an emission wavelength of 535 nm.

### Western Blot (WB) Detection of RANK Expression

Samples were lysed on ice for 10 min and then centrifuged at 4 °C at 14,000 g for 15 min. After quantifying protein concentrations using a BCA protein quantification kit, 80 μL of protein was mixed with 20 μL of 5 × protein loading buffer and boiled for 5 min in a boiling water bath. SDS-PAGE electrophoresis was performed. After transferring to PVDF membranes, the PVDF membrane was taken out, and the cut strips were placed in a sealing solution (5% skim milk) at room temperature for 40 min on a shaker. The primary antibody RANK (invitrogen, MA5-16153) was incubated overnight at 4 °C, followed by a 40-min incubation with the secondary antibody, signal development, and image capture. WB band quantification analysis was performed using Image J (version 1.8.0.345) software, where the relative quantification of the protein was calculated by dividing the grayscale value (max-mean) of the target protein by the grayscale value of the internal reference protein.

### Statistical analysis

Statistical analysis was conducted using Prism GraphPad. Data obtained from the experiments were presented as mean ± standard error of the mean (Mean ± SEM). For comparisons between groups of quantitative data, *t*-tests(Dunnet-t test was used for pairwise comparisons) were used, and for comparisons among multiple groups of quantitative data, one-way analysis of variance (ANOVA) was performed. A significance level of *P* < 0.05 indicated statistical significance, *P* < 0.01 indicated significant statistical difference, and *P* < 0.001 indicated highly significant statistical difference.

## Results

### Joint metabolome-16S analysis reveals a significant decrease in lactic acid bacteria and C4 in postmenopausal osteoporosis (PMOP)

**Figure 1:**
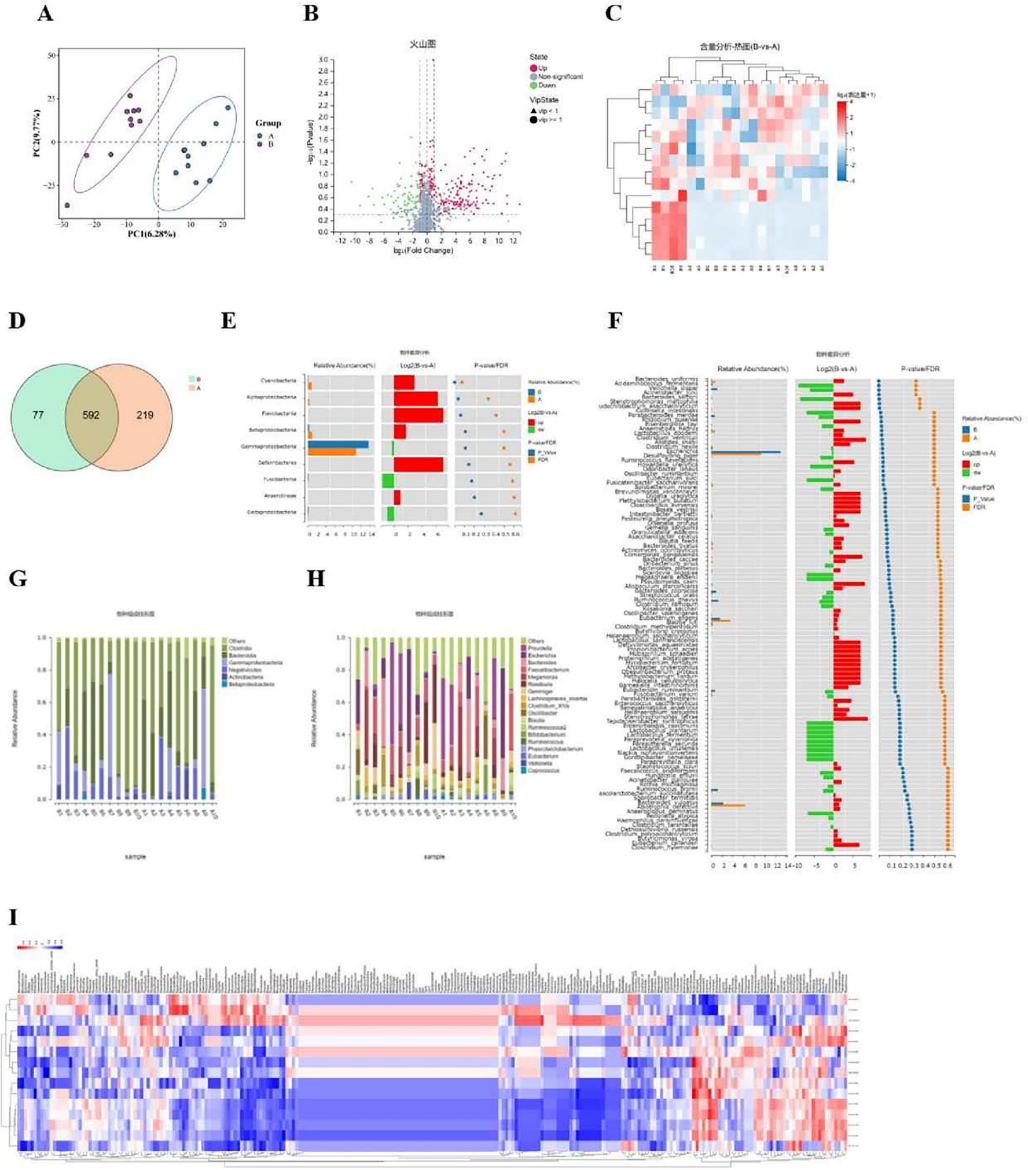
Clinical Evidence Demonstrating La-02 and C4 as Key Factors in Mediating PMOP (**A**) PLSDA Plot: The horizontal axis represents X-variate1, i.e., the first principal component, with the percentage of explanation in parentheses. The vertical axis represents X-variate2, i.e., the second principal component, with the percentage of explanation in parentheses; (**B**) Volcano Plot: The horizontal axis displays the log2-transformed Fold Change, while the vertical axis shows the -log10 converted q-value. In the plot, significantly downregulated metabolites are depicted in blue, significantly upregulated metabolites are in red, and non-significant metabolites are shown in gray; (**C**) Content Analysis-Heatmap: Each row in the heatmap represents a differentially expressed metabolite, and each column represents a sample. The color scale represents expression levels, ranging from blue (low) to red (high); (**D**) Venn Diagram: Different colored shapes in the diagram represent different samples or groups, and the overlapping numbers indicate the number of shared OTUs between two samples or groups; (**E**) Species Differential Wilcox Test results diagram: The left side displays the relative abundance bar chart of species in each group. The middle section represents the log2-transformed average relative abundance ratio of the same species between two groups. The right side shows the P-value and FDR values obtained using the Wilcox test. (**F**) Phylum-Level Differential Species Analysis; (**G**) Phylum-Level Species Composition Bar Chart: The x-axis represents sample names, and the y-axis shows the relative abundance of annotated species; (**H**) Genus-Level Species Composition Bar Chart; (**I**) Spearman Correlation Heatmap between Genus Microbial Taxa and Differential Metabolites: Each column represents a differential metabolite, and each row represents a microbial taxon. The symbols * represent *P* < 0.05, and ** represent *P* < 0.01.

To investigate potential biomarkers involved in postmenopausal osteoporosis (PMOP) at the level of small biological molecules, we conducted a joint metabolome-16S analysis on human serum and fecal samples. The results of non-targeted metabolomics sequencing showed that, in comparison to the healthy control group, a total of 15 significantly different metabolites were identified in PMOP patients, with 12 upregulated and 3 downregulated. Notably, butyric acid (C4) exhibited the most significant downregulation in PMOP patients’ blood (Fig. 1A–C). Non-targeted metabolomics sequencing results also revealed a significant decrease in the abundance of Lactic Acid Bacteria in the top 10 most abundant species. Furthermore, the content of La-02 was significantly reduced in the feces of PMOP patients (Fig. 1D–H). By performing joint analysis of 16S rDNA and non-targeted metabolomics, it was discovered that La-02 was positively correlated with C4 (Fig. 1I), and both were significantly downregulated in PMOP patients.

### La-02 and C4 improve OVX-induced pathological changes

**Figure 2:**
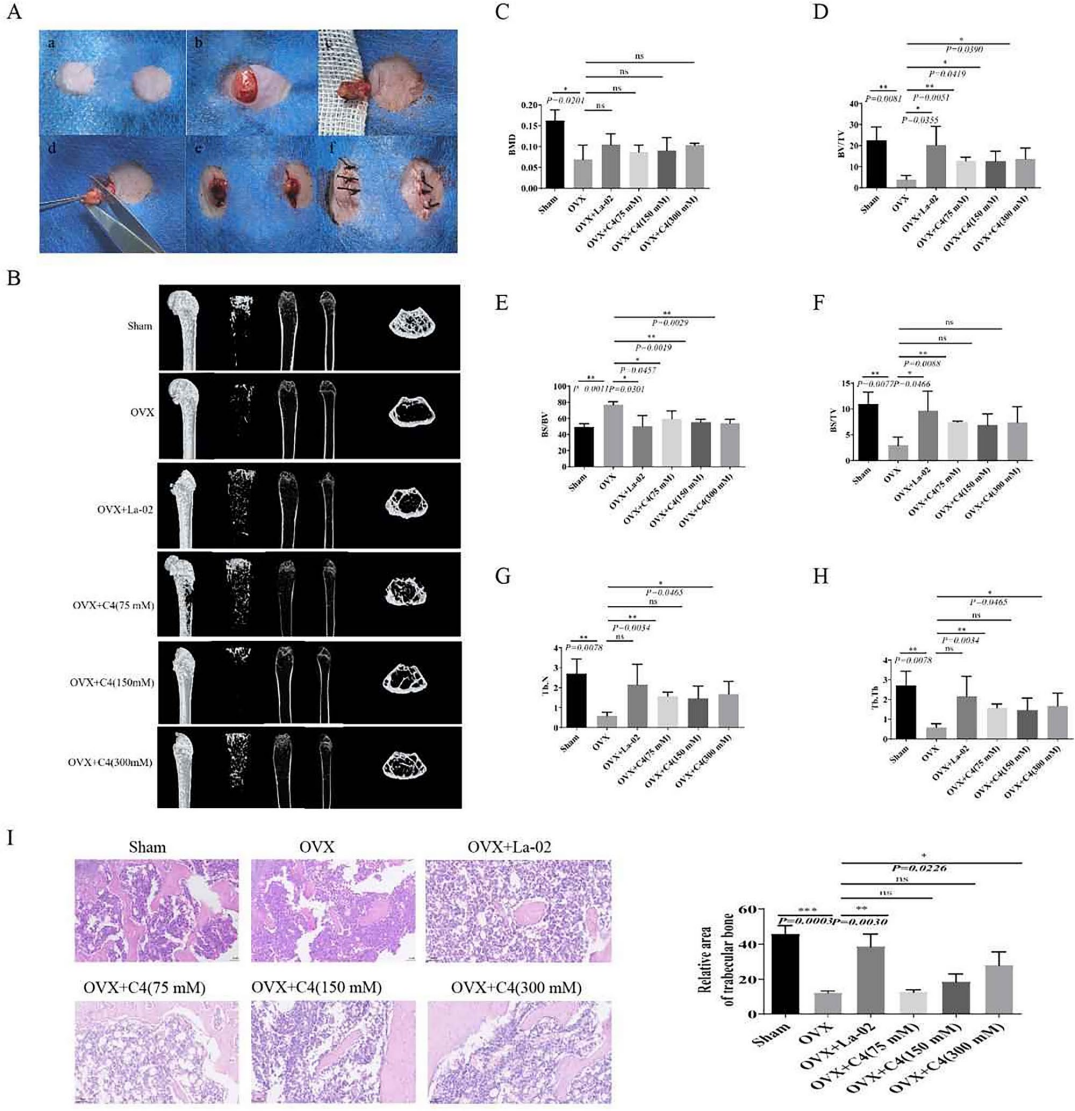
La-02 and C4 Improve Postmenopausal Osteoporosis (**A**) Ovariectomy (OVX) was performed on mice to induce a postmenopausal osteoporosis model. Mice were subjected to either 108 CFU/mL La-02 via gavage for 2 weeks or low, medium, and high concentrations of C4 for 8 weeks; (**B**) Representative 3D-μCT images of the left femur; (**C**) Bone mineral density (BMD); (**D**) Bone volume/total volume (BV/TV); (**E**) Bone surface/bone volume (BS/BV); (**F**) Bone surface/total volume (BS/TV); (**G**) Trabecular number (Tb.N); (**H**) Trabecular thickness (Tb.Th); (**I**) HE staining to detect bone loss (200X). * represents *P* < 0.05, ** represents *P* < 0.01, *** represents *P* < 0.001, **** represents *P* < 0.0001.

Using female mice as subjects, osteoporosis was induced by bilateral ovariectomy (OVX) (Fig. 2A). After intervention with La-02 and C4, the bone structural characteristics of the left femur in mice were analyzed through Micro-CT scanning. The 3D-μCT image is shown in Fig. 2B. In the La-02 and C4 groups, bone volume/total volume (BV/TV) (Fig. 2D), bone surface/total volume (BS/TV) (Fig. 2F), trabecular number (Tb.N) (Fig. 2G), and trabecular thickness (Tb.Th) (Fig. 2H) all significantly increased, while bone surface/bone volume (BS/BV) (Fig. 2E) significantly decreased. Bone mineral density (BMD) (Fig. 2C) did not show significant changes. Similarly, HE staining results indicated that La-02 and high-concentration C4 intervention significantly reduced the loss of trabeculae caused by estrogen deprivation (Fig. 2I). These results suggest that La-02 and high-concentration C4 can significantly improve bone loss in mice after OVX.

### La-02 and C4 improve intestinal permeability in OVX mice

**Figure 3:**
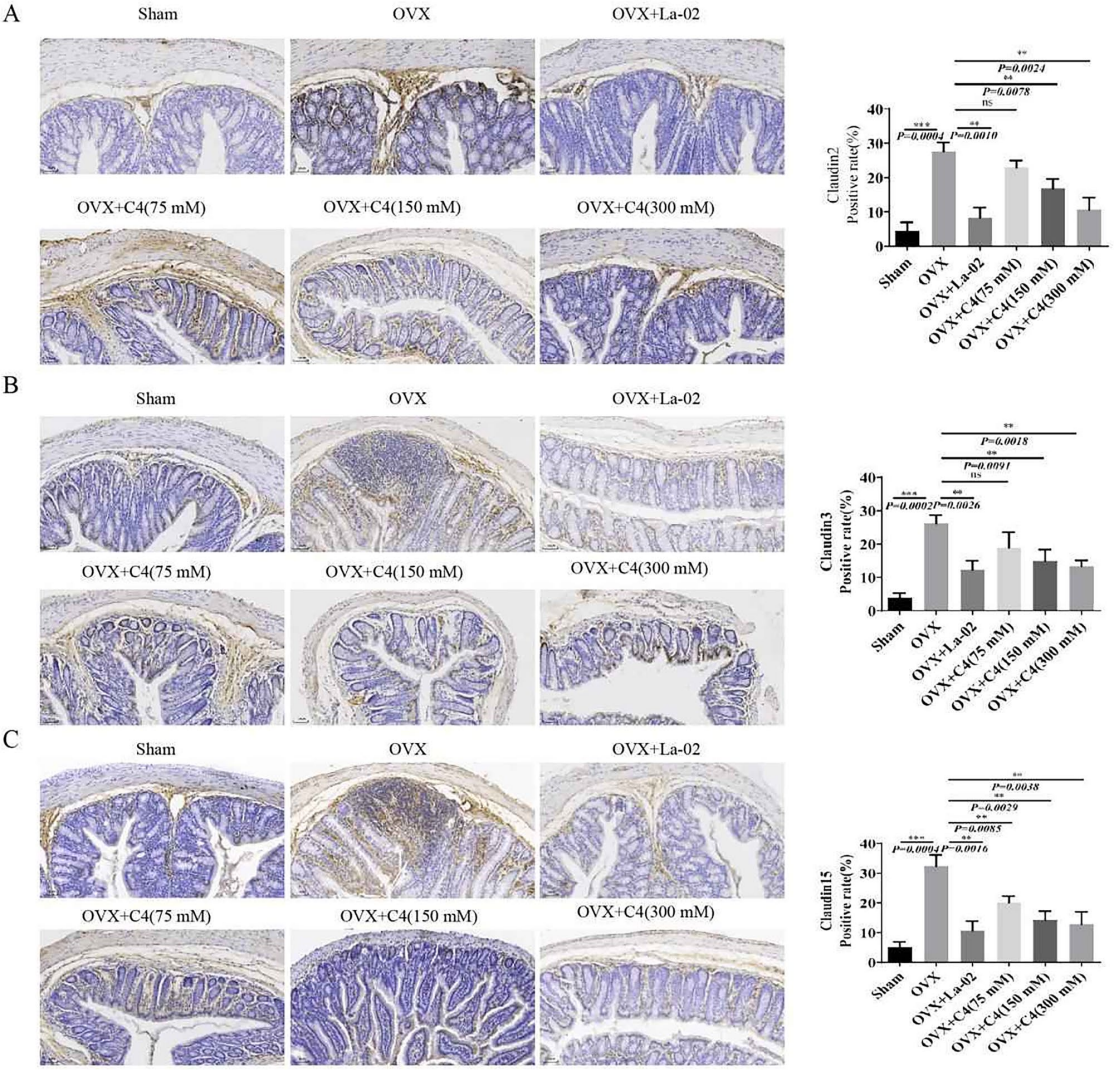
La-02 and C4 Improve Intestinal Permeability (**A**) IHC detection of Claudin 2 expression in colonic tissue (200X magnification); (**B**) IHC detection of Claudin 3 expression in colonic tissue (200X); (**C**) IHC detection of Claudin 15 expression in colonic tissue (200X). *represents *P* < 0.05, ** represents *P* < 0.01, *** represents *P* < 0.001, **** represents *P* < 0.0001.

An intact intestinal barrier plays a crucial role in preventing the abnormal transport of harmful toxins and is vital for maintaining metabolic balance in the body. We used IHC to assess the impact of La-02 and C4 on the protein levels of Claudin family members in the intestine, which are key constituents of tight junction proteins that maintain intestinal barrier integrity. The results showed that, compared to the Sham group, the expression of Claudin 2, Claudin 3, and Claudin 15 in the colon tissue of the OVX group significantly increased. However, after intervention with La-02 and C4, the expression of Claudin 2 (Fig. 3A), Claudin 3 (Fig. 3B), and Claudin 15 (Fig. 3C) in the colon tissues significantly decreased. This suggests that La-02 and C4 not only inhibit bone loss in OVX mice but also improve their intestinal permeability.

### La-02 and C4 suppress osteoclastogenesis and bone resorption

**Figure 4:**
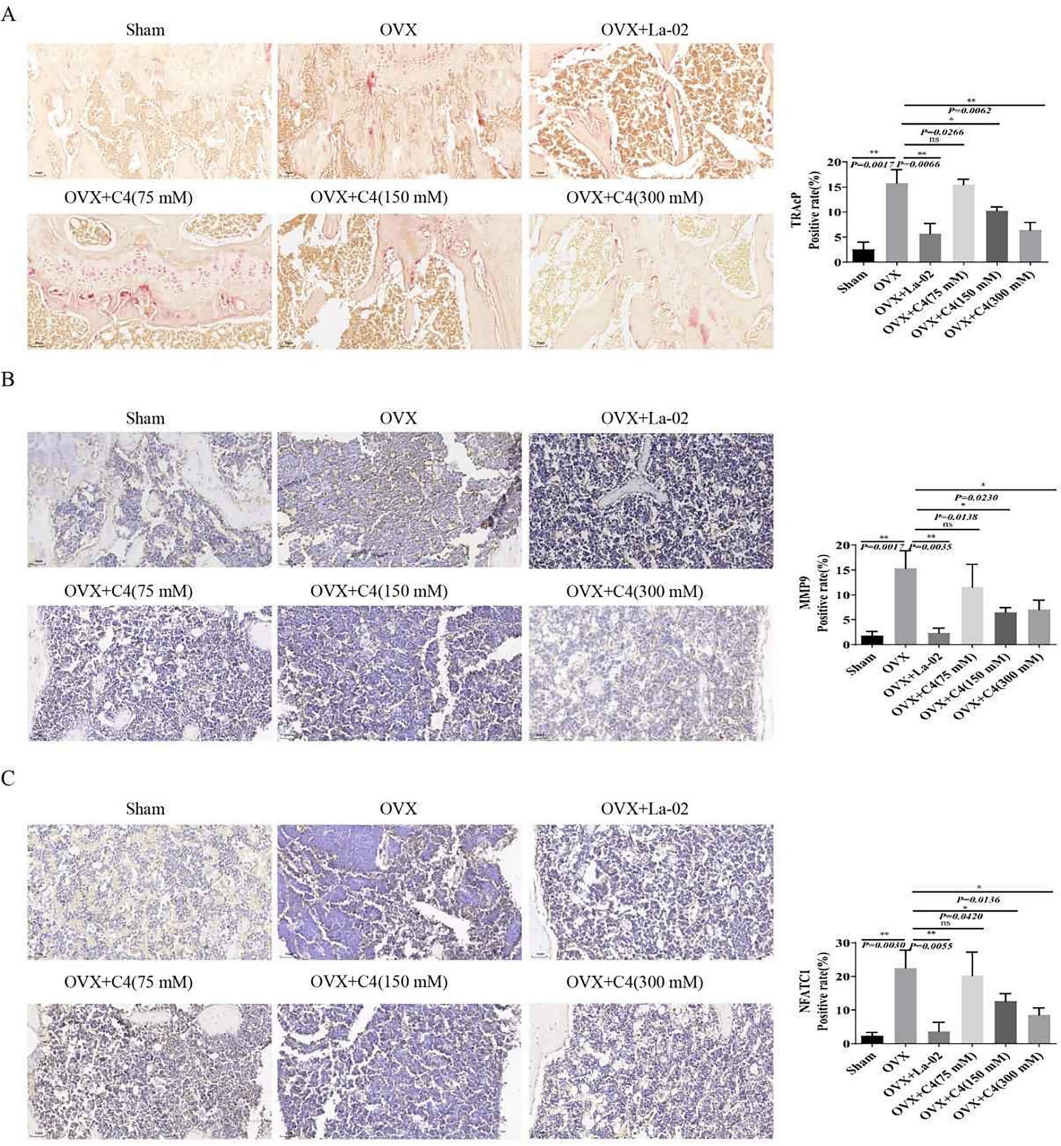
La-02 and C4 Inhibit Osteoclast Formation and Bone Resorption Activity (**A**) TRAcP activity staining to detect the number of osteoclasts (200X); (**B**) Immunohistochemical (IHC) staining for the expression of bone resorption marker MMP9 (200X); (**C**) Immunohistochemical (IHC) staining for the expression of bone resorption marker NFATc1 (200X). * represents *P* < 0.05, ** represents *P* < 0.01, *** represents *P* < 0.001, **** represents *P* < 0.0001.

To investigate the effects of La-02 and C4 on osteoclastogenesis and bone resorption, tartrate-resistant acid phosphatase (TRAcP) activity staining was used to detect the number of osteoclasts. The results showed that compared to the Sham group, the OVX group exhibited significant increases in osteoclast formation markers TRAcP, bone resorption marker protein MMP9, and NFATc1. However, both La-02 and high-concentration C4 interventions significantly inhibited osteoclast formation (Fig. 4A). Additionally, the expression of MMP9 (Fig. 4B) and NFATc1 (Fig. 4C) were significantly reduced after La-02 and high-concentration C4 interventions. These results suggest that in vivo, La-02 and high-concentration C4 can suppress osteoclast formation and bone resorption.

### La-02 and C4 suppress B-cell RANKL expression

**Figure 5:**
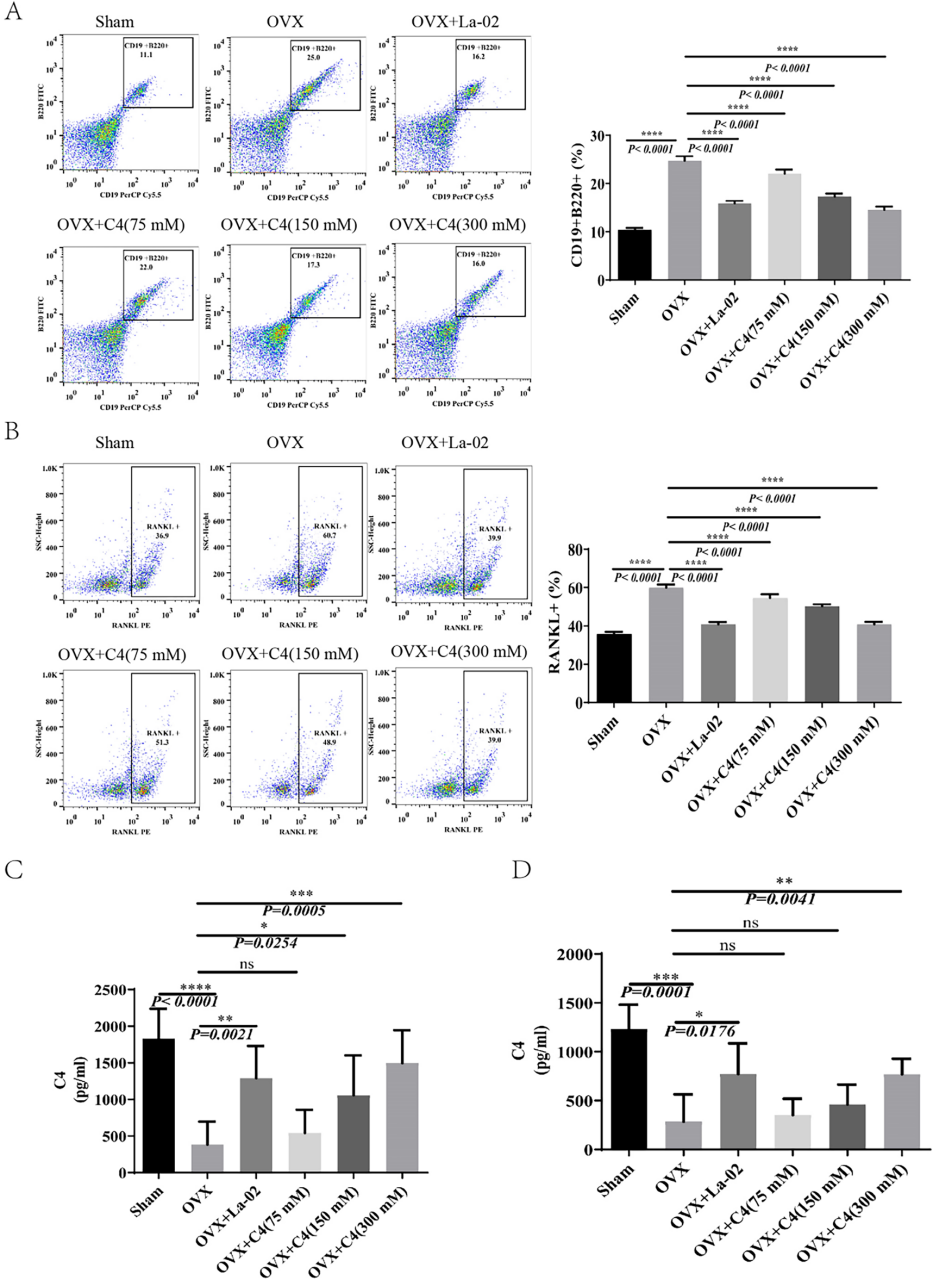
La-02 and C4 Inhibit B-Cells and B-Cell RANKL Production (**A**) Flow cytometric analysis of the quantity of B cells in the bone marrow; (**B**) Flow cytometric analysis of changes in RANKL expression on B cells in the bone marrow; (**C**)Measurement of C4 levels in the feces of OVX mice using assay kits; (**D**) Measurement of C4 levels in the serum of OVX mice using assay kits. * represents *P* < 0.05, ** represents *P* < 0.01, *** represents *P* <0.001, **** represents *P* < 0.0001.

To investigate whether La-02 and C4 can affect B-cell activity, we used flow cytometry to measure the content of mature B lymphocytes (CD5−CD19+B220+) in the bone marrow of OVX mice. The results showed a significant reduction in CD5−CD19+B220+cells after La-02 and C4 interventions (Fig. 5A). Additionally, we also assessed the content of RANKL+ cells within mature B lymphocytes in the bone marrow. The results indicated a significant decrease in RANKL+ cells after La-02 and C4 interventions (Fig. 5B). Finally, we validated whether La-02 could affect the expression levels of C4. Using assay kits, we measured the expression of C4 in the serum and feces of OVX mice. The results showed a significant increase in C4 expression levels in feces after La-02 intervention (Fig. 5C), and a significant increase in serum C4 levels as well (Fig. 5D). This suggests that La-02’s inhibitory effects on bone loss, intestinal permeability, RANKL+ B-cell content, osteoclast formation, and bone resorption in OVX mice may be mediated through the regulation of C4 levels.

### In vitro validation of La-02 and C4 in inhibiting bone resorption

**Figure 6:**
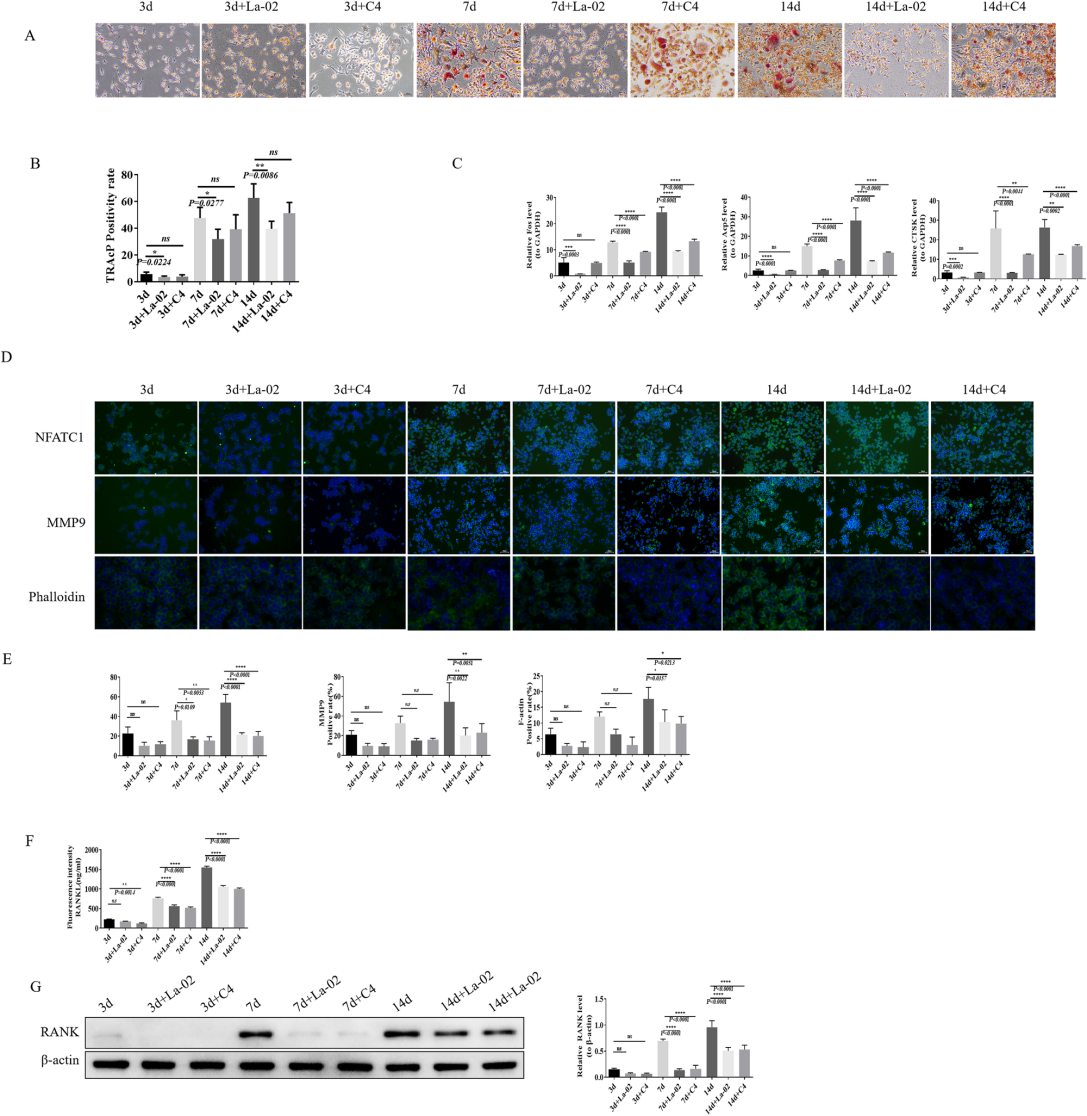
In Vitro Validation of the Inhibitory Effects of La-02 and C4 on Osteoclast Differentiation (**A**) TRAcP staining to assess osteoclast differentiation; (**B**) Statistical analysis of TRAcP staining results; (**C**) RT-PCR to measure the expression levels of osteoclast-related genes CTSK, Acp5, and c-Fos; (**D**) IF staining for the expression of osteoclast-related proteins; (**E**) Statistical analysis of IF staining results; (**F**) Assay kit to evaluate osteoclast bone resorption ability; (**G**) WB to measure RANK expression, with β-actin as the internal reference protein. * represents *P* < 0.05, ** represents *P* < 0.01, *** represents *P* < 0.001, **** represents *P* < 0.0001.

RAW264.7 was cultured in vitro and osteoclast differentiation factor (RANKL) was applied to induce RAW264.7 cells into osteoclasts. In the process of inducing differentiation, La-02 supernatant and C4 were used to intervene in a certain ratio, and the osteoclast content was detected by TRAcP staining on days 3, 7, and 14, respectively, and the results showed that both La-02 and C4 significantly inhibited the formation of osteoclasts (Fig. 6A,B). Meanwhile, RT-PCR was used to detect the expression levels of genes related to osteoclastogenesis, and the results showed that La-02 and C4 significantly inhibited the expression of CTSK, Acp5 and c-Fos (Fig. 6C). Finally, IF experiments were conducted to detect the expression of F-actin (F-actin ring is a prominent feature of mature osteoclasts) and bone resorption markers MMP9 and NFATc1. La-02 and C4 were found to inhibit the expression of F-actin, MMP9, and NFATc1 (Fig. 6D,E). Osteoclast bone resorption ability was also assessed using an assay kit, yielding similar results (Fig. 6F). Lastly, western blotting was performed to measure the expression levels of RANK on osteoclasts, and it was observed that La-02 and C4 significantly inhibited RANK expression on osteoclasts (Fig. 6G). This suggests that the improvement by La-02 in OVX may be achieved through the regulation of C4 levels, leading to the suppression of RANKL+ B cells and subsequent inhibition of osteoclast formation.

## Discussion

Postmenopausal osteoporosis (PMOP) is a systemic disease that poses a serious threat to the health of postmenopausal women^22^, leading to a reduced quality of life. Traditional anti-osteoporosis medications have certain limitations. On one hand, long-term drug therapy can increase the risk of rare side effects, especially with bone resorption inhibitors (such as osteonecrosis of the jaw and kidney damage). On the other hand, some anti-osteoporosis drugs result in a rapid decrease in efficacy once discontinued (e.g., denosumab). Most patients have low acceptance of existing treatments, low long-term treatment compliance, and may not be able to afford the treatment costs. Exploring new treatment approaches is urgently needed. Previous research has indicated that imbalances in the gut microbiota resulting from factors such as diet, medications, and inflammation can impact bone health^7^. Evaluating the effectiveness of probiotics, prebiotics, or microbial metabolites in treating osteoporosis has become a hot topic in research. The gut microbiota and its metabolic products, as a relatively “natural” approach to treatment, offer certain therapeutic advantages and promising prospects.

Using 16SrDNA combined with non-target metabolome sequencing is one of the common methods to explore intestinal microorganisms and metabolites^23–25^.In this study, we employed 16S rDNA combined with non-targeted metabolomics sequencing to investigate probiotics and their metabolites in the feces of clinical PMOP patients and explored the relationships between them. The 16S rDNA sequencing results revealed decreased abundance of several bacterial genera, including Prevotella, Lactobacillus Beijerinck, Faecalibacterium, Bacteroides, Megamonas, Lachnospiracea, and Oscillibacter in PMOP patients. Notably, the abundance of the Lactobacillus Beijerinck was significantly reduced. Similarly, several studies assessing gut bacteria quantity and diversity in osteoporosis patients have shown a significant decrease in gut bacterial diversity. Some species such as Dialister, Faecalibacterium, and Tolumonas increased, while Bacteroides and Roseburia decreased significantly, along with a substantial reduction in Lactobacillus, This is consistent with our experimental results^26–29^. Our study showed that among the Lactobacillus genus, *Lactobacillus acidophilus* had the most significant decrease, suggesting that the downregulation of *L. acidophilus* in the gut may be one of the primary factors contributing to bone loss in PMOP patients. Research by Yuan et al. also observed a decreased abundance of *L. acidophilus* within the Firmicutes phylum in osteoporotic mice^30^. Non-targeted metabolomics sequencing results showed significant reductions in several metabolites, including Butyric acid, Androsterone sulfate, and 3-(4-methoxyphenyl) propyl hydrogen sulfate in PMOP patients, with Butyric acid being particularly significantly reduced. To explore the relationship between gut microbial communities and host metabolic phenotypes, we calculated Spearman correlation coefficients between microbes and metabolites. The results showed a positive correlation between Lactobacillus and butyric acid. This is consistent with the study results of Xie et al. The total SCFAs and butyric acid in the serum of piglets were significantly increased after *L. acidophilus* intervention^31^. Similarly, research by Chen et al. revealed that the diversity of gut microbial communities in osteoporosis patients decreased with reduced abundances of Lactobacillus and butyric acid-producing bacteria. Furthermore, in vitro experiments showed that *L. acidophilus* and xylooligosaccharide Lactobacillus supernatants and their metabolite butyric acid promoted the proliferation, differentiation, and maturation of osteoblasts^32^. These research findings collectively suggest that *L. acidophilus* and butyric acid may be key biomarkers involved in mediating PMOP. Exploring the associations between *L. acidophilus*, butyric acid, and the development of PMOP, as well as their potential mechanisms of action, has piqued our interest.

To investigate the impact of *L. acidophilus* on bone metabolism, this study conducted interventions with *L. acidophilus* in an OVX mouse model. The results showed that *L. acidophilus* intervention improved bone density and reduced bone loss caused by estrogen withdrawal, indicating its potential in ameliorating osteoporosis. Chen et al. have previously demonstrated that *L. acidophilus* can influence bone metabolism by promoting osteoblast proliferation^33^, and Osteoblasts (bone formation) and osteoclasts (bone resorption) are two important factors in the exploration of osteoporosis^33^. However, whether *L. acidophilus* also affects osteoclasts is not yet reported. Our experimental findings revealed an increase in bone trabeculae following *L. acidophilus* intervention, suggesting that *L. acidophilus* might inhibit osteoclast formation and, consequently, bone resorption. To validate this hypothesis, we assessed osteoclast activity in OVX mice after *L. acidophilus* intervention. The results indicated that *L. acidophilus* significantly inhibited osteoclast formation and bone resorption. These results confirm that *L. acidophilus* can improve PMOP by suppressing osteoclast formation. However, further research is needed to elucidate the specific mechanisms by which *L. acidophilus* inhibits osteoclast formation.

Numerous studies have reported that probiotics primarily influence bone metabolism through their metabolic byproducts^11^. Previous experimental findings suggested that *L. acidophilus* might improve PMOP by mediating the production of butyric acid. Therefore, we conducted tests to measure the levels of butyric acid in the serum and feces of OVX mice after *L. acidophilus* intervention. The results showed a significant increase in butyric acid content following *L. acidophilus* intervention, indicating that butyric acid could be a key mediator through which *L. acidophilus* exerts its effects. To further investigate this hypothesis, we administered different concentrations of butyrate to OVX mice. The results demonstrated that butyrate significantly inhibited osteoclast formation and improved PMOP, with higher concentrations of butyrate showing particularly pronounced effects. These experimental findings suggest that *L. acidophilus* can inhibit osteoclast formation through butyric acid, and the extent of inhibition is closely related to the concentration of butyric acid. However, the mechanisms by which butyric acid inhibits osteoclast formation still require further exploration.

There is literature reporting that short-chain fatty acids (SCFAs), primarily acetate, propionate, and butyrate, can impact bone metabolism by directly affecting cells involved in bone metabolism, such as osteoblasts, osteoclasts, and chondrocytes, or indirectly by inducing the production of certain anti-inflammatory factors and immune-regulatory responses, thereby contributing significantly to bone healing^34,35^.

It has been documented that SCFAs play a crucial role in the complex biochemical interactions involving osteoclasts. One key pathway through which SCFAs influence osteoclasts is by regulating the immune system’s response, subsequently affecting osteoclasts^36^. Tyagi et al. reported that butyrate can regulate bone metabolism by inducing the production of Wnt10b on CD8+ T cells mediated by Treg cells. Begka et al. also confirmed that butyrate can modulate the immune system and impact bone metabolism^37^. Butyrate salt-mediated Treg cell induction can prevent osteoclast formation through mechanisms involving the secretion of anti-osteoclast factors like IL-10 and CTLA4/CD80/86 cell–cell contact-dependent pathways. However, studies on Rag1 knockout mice (lacking T and B cells) still showed increased bone density after butyrate treatment, suggesting that butyrate has a direct inhibitory effect on osteoclasts^17^. These studies collectively suggest that the inhibition of osteoclast formation mediated by butyrate is likely the result of multiple pathways, including direct inhibition, immune-related signaling, and metabolic changes.

The literature reports that B cells play a significant role in the regulation of osteoclast formation through the RANKL-OPG signaling system^22^. Onal M’s research has demonstrated that the expression of RANKL on B cells, and its binding to RANK on osteoclasts, contributes to the increase in osteoclast numbers^38^.

To investigate whether butyric acid inhibits the expression of RANKL on B cells and osteoclasts, we examined the mature B lymphocytes in the bone marrow of OVX mice and their expression of RANKL after several weeks of butyrate intervention. The results showed that butyrate intervention significantly inhibited the expression of B cells and RANKL on B cells. Additionally, we also measured the expression levels of RANK on osteoclasts, and the results showed that butyrate significantly inhibited the expression of RANK on osteoclasts. These experimental results suggest that butyrate may inhibit the formation of the RANKL-RANK axis by suppressing RANKL on B cells and RANK on osteoclasts, thereby inhibiting osteoclast formation. Furthermore, butyric acid can directly inhibit the production of RANK on osteoclasts, indicating that butyric acid may directly suppress osteoclast formation. To confirm this hypothesis, we intervened with both *L. acidophilus* and butyric acid during the induction of osteoclast precursor cell differentiation. The results showed that *L. acidophilus* and butyric acid directly inhibited osteoclast formation and bone resorption activity. These experimental results suggest that the mechanism by which butyric acid inhibits osteoclast formation is diverse, involving both direct suppression of osteoclast formation and mediation through the immune system.

Other studies have reported that restoring and maintaining the intestinal epithelial barrier, enhancing gut barrier function, is an essential part of gastrointestinal immunity^39^. A healthy intestinal epithelial barrier can prevent hyperpermeability caused by impaired tight junctions^40^. Hyperperperpermeability or “leaky gut” can lead to the efflux of abnormally high levels of inflammatory cytokines, resulting in systemic inflammation and excessive activity of osteoclasts, leading to bone degradation^41^. Therefore, we also examined the effect of *L. acidophilus* and butyrate on the protein levels of Claudin family members in the intestine. The results showed that both *L. acidophilus* and butyrate intervention significantly increased the expression of claudin 2, claudin 3, and claudin 15 in colon tissues, indicating that *L. acidophilus* and butyrate intervention improved intestinal permeability and indirectly affected bone metabolism.

In this study, we have confirmed that *L. acidophilus* and butyric acid can improve PMOP through various pathways, including improving the intestinal barrier to prevent the transport of harmful toxins and biomolecules, promoting osteoblast activity, and mediating the immune system to affect bone metabolism. pathway, which is consistent with existing research results, but it has not been reported that *L. acidophilus* inhibits the expression of RANKL on B cells by affecting the amount of its metabolite butyrate, thereby inhibiting the formation of the RANKL-RANK axis and thus inhibiting osteoclastogenesis. However, whether butyric acid directly inhibits osteoclasts or indirectly suppresses osteoclast formation by inhibiting RANKL expression on B cells, the underlying mechanisms remain unclear. In future research, we plan to explore the key genes and signaling pathways involved in butyric acid’s direct inhibition of osteoclast activity and its impact on B cell RANKL production using transcriptome sequencing. We will also elucidate the mechanisms of butyric acid’s direct and indirect inhibition of osteoclast activity through in vivo and in vitro rescue experiments. Additionally, we will investigate which pathway is the primary mechanism through which butyric acid improves PMOP using co-culture experiments in vitro. In summary, this study provides a theoretical basis for the clinical application of *L. acidophilus* and butyric acid in the treatment of postmenopausal osteoporosis (PMOP). It aims to offer safer and more cost-effective treatment options for PMOP patients.

### Supplementary Information

Supplementary Information 1.

Supplementary Information 2.

Supplementary Information 3.

Supplementary Information 4.

Supplementary Information 5.

Supplementary Information 6.

## Data Availability

All data and material are available from the corresponding author upon reasonable request.
