# Supplementary figures and images for: Mechanistic study on the alleviation of postmenopausal osteoporosis by Lactobacillus acidophilus through butyrate-mediated inhibition of osteoclast activity
Source: Sci Rep. 2024 Mar 25;14:7042. doi: 10.1038/s41598-024-57122-x (PMC10963762; doi:10.1038/s41598-024-57122-x)

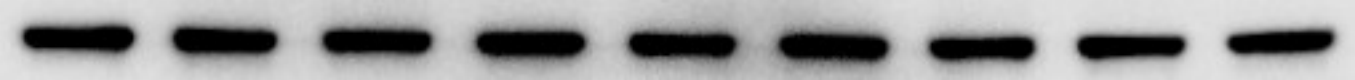

Supplement: Supplementary file 1 — Supplementary Information 1. [file 41598_2024_57122_MOESM1_ESM.pdf]

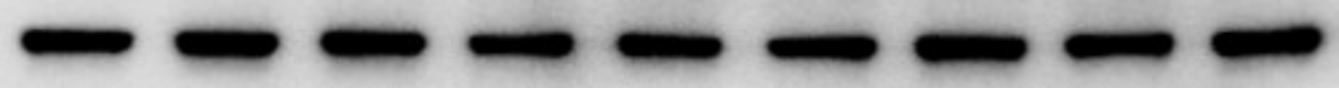

Supplement: Supplementary file 2 — Supplementary Information 2. [file 41598_2024_57122_MOESM2_ESM.pdf]

CtSK

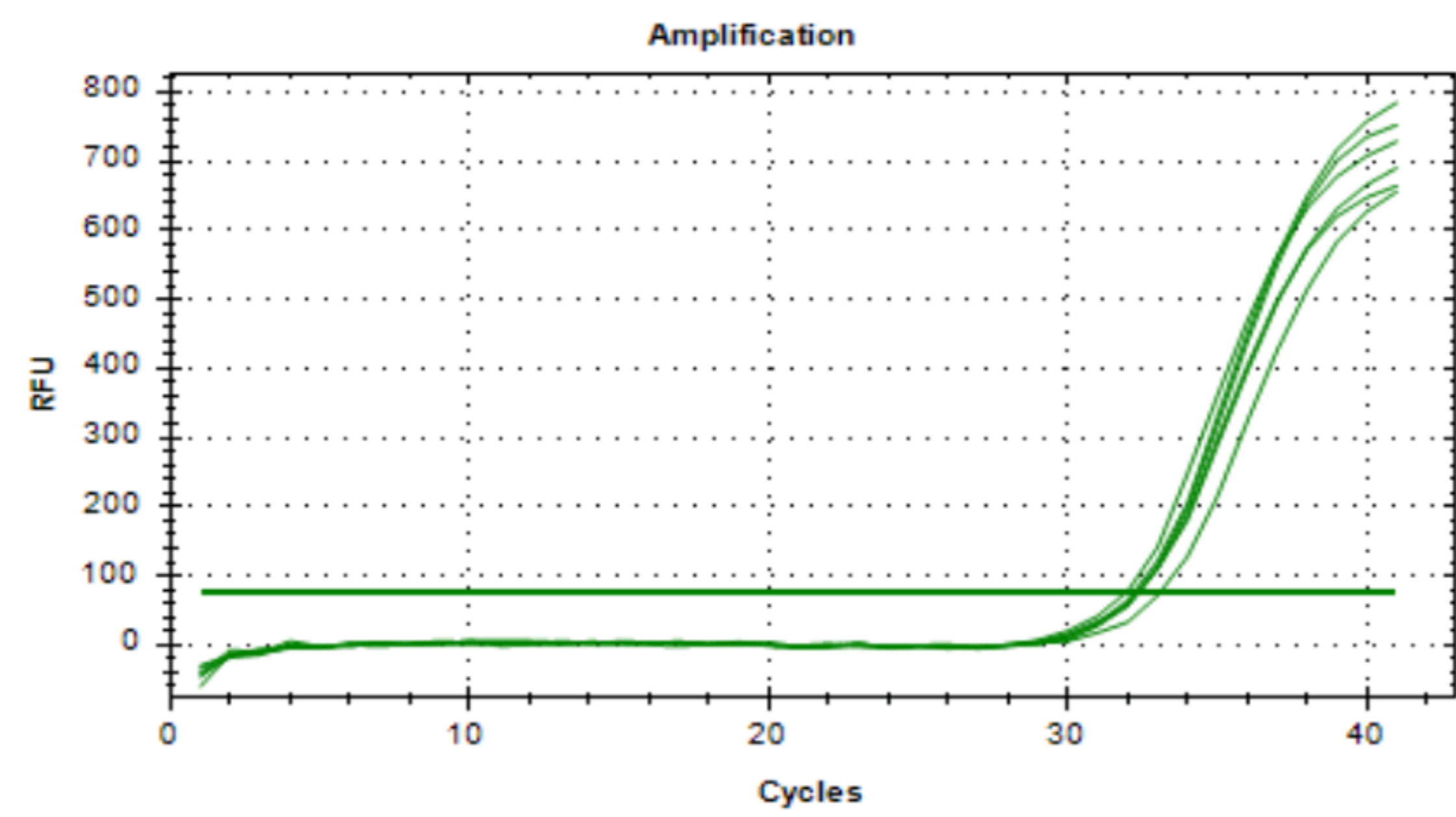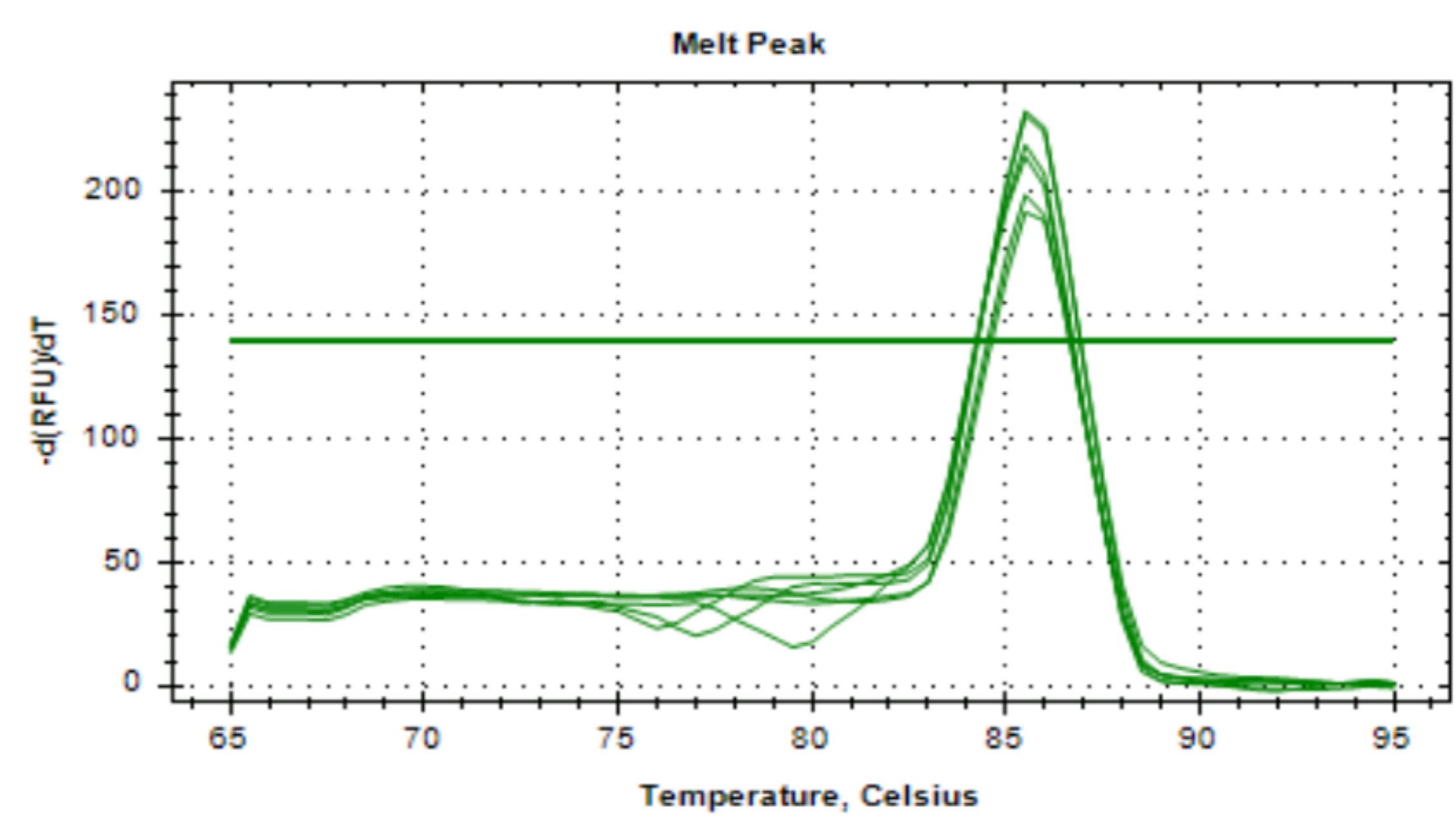

ACPS

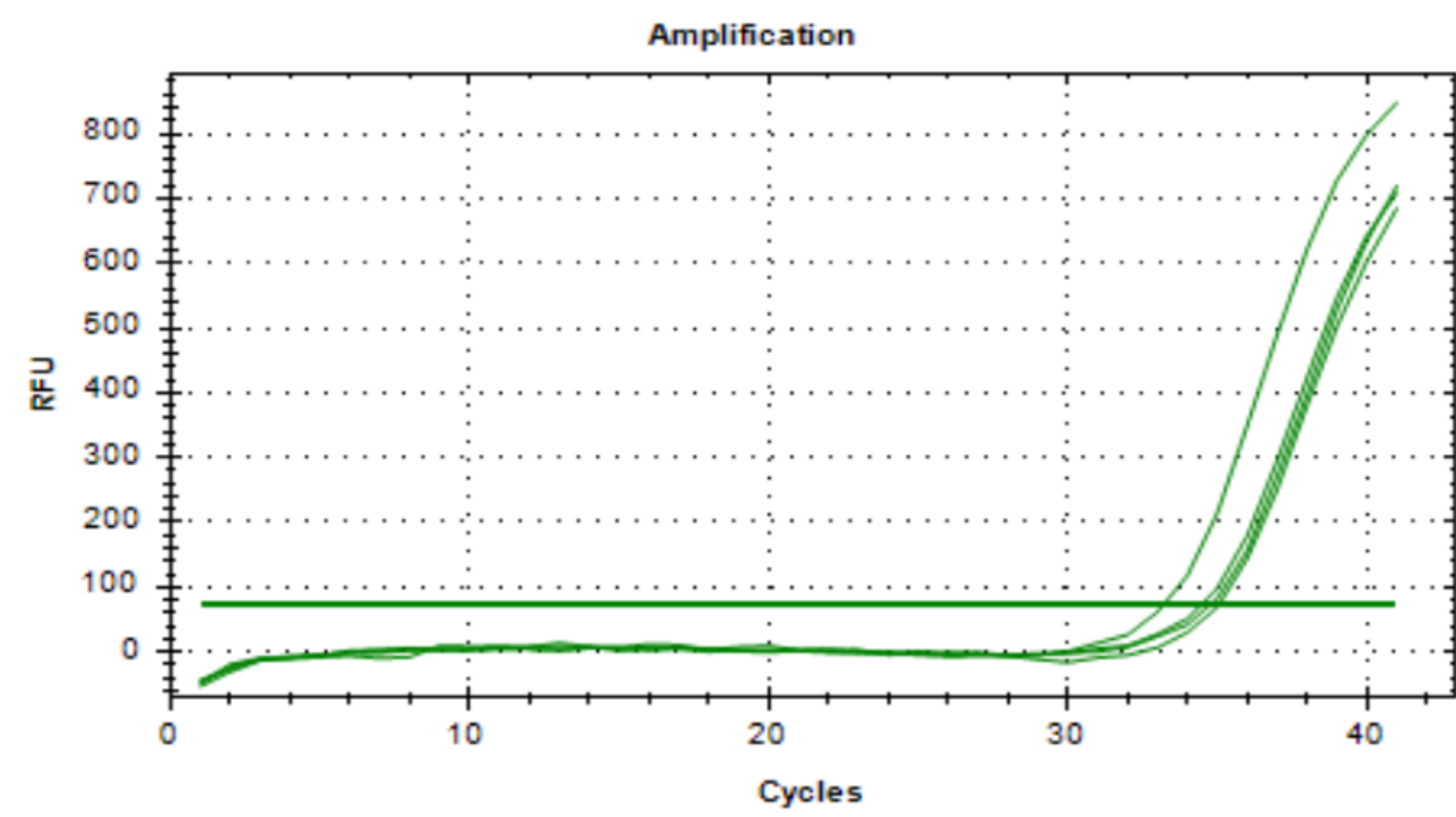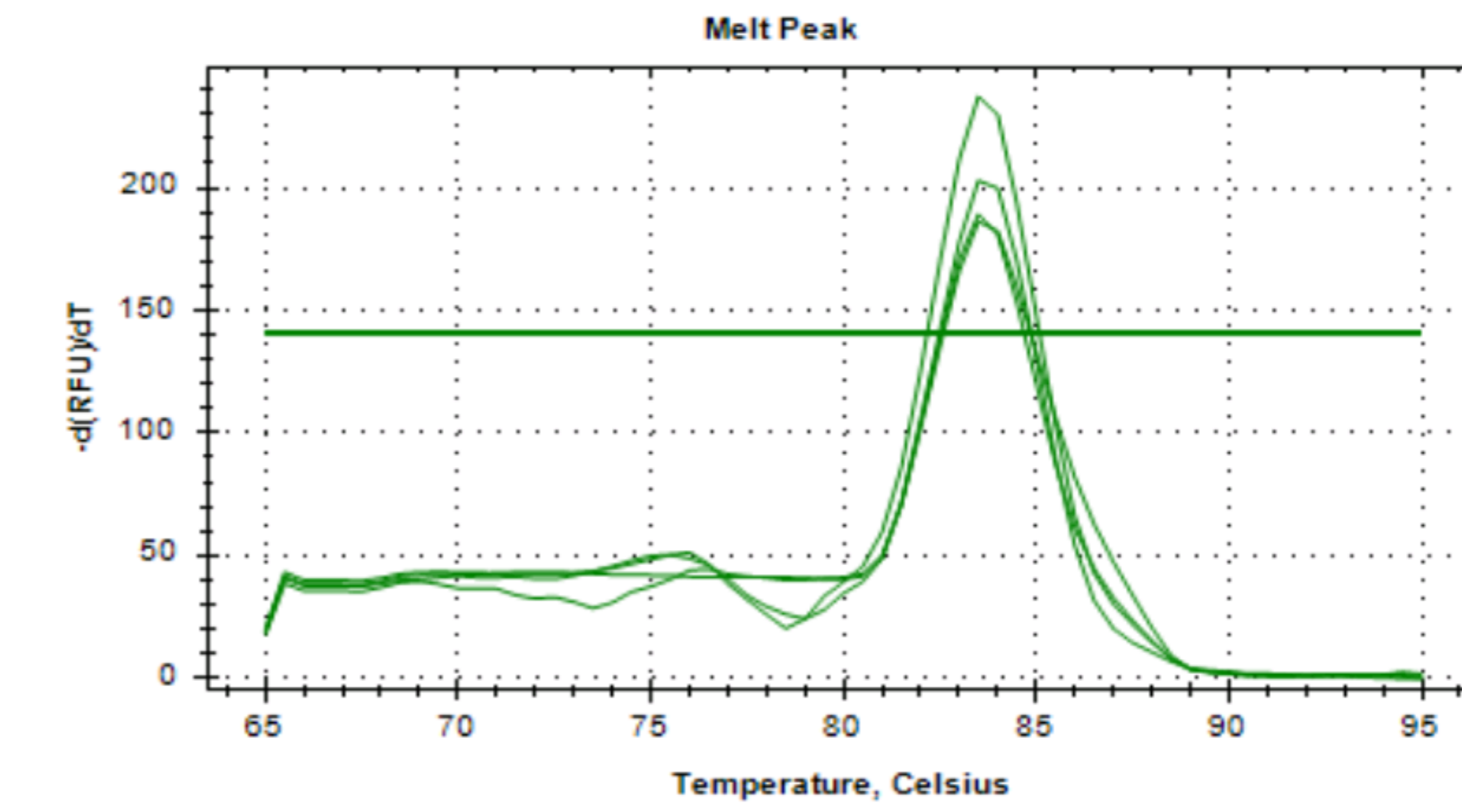

C-FOS

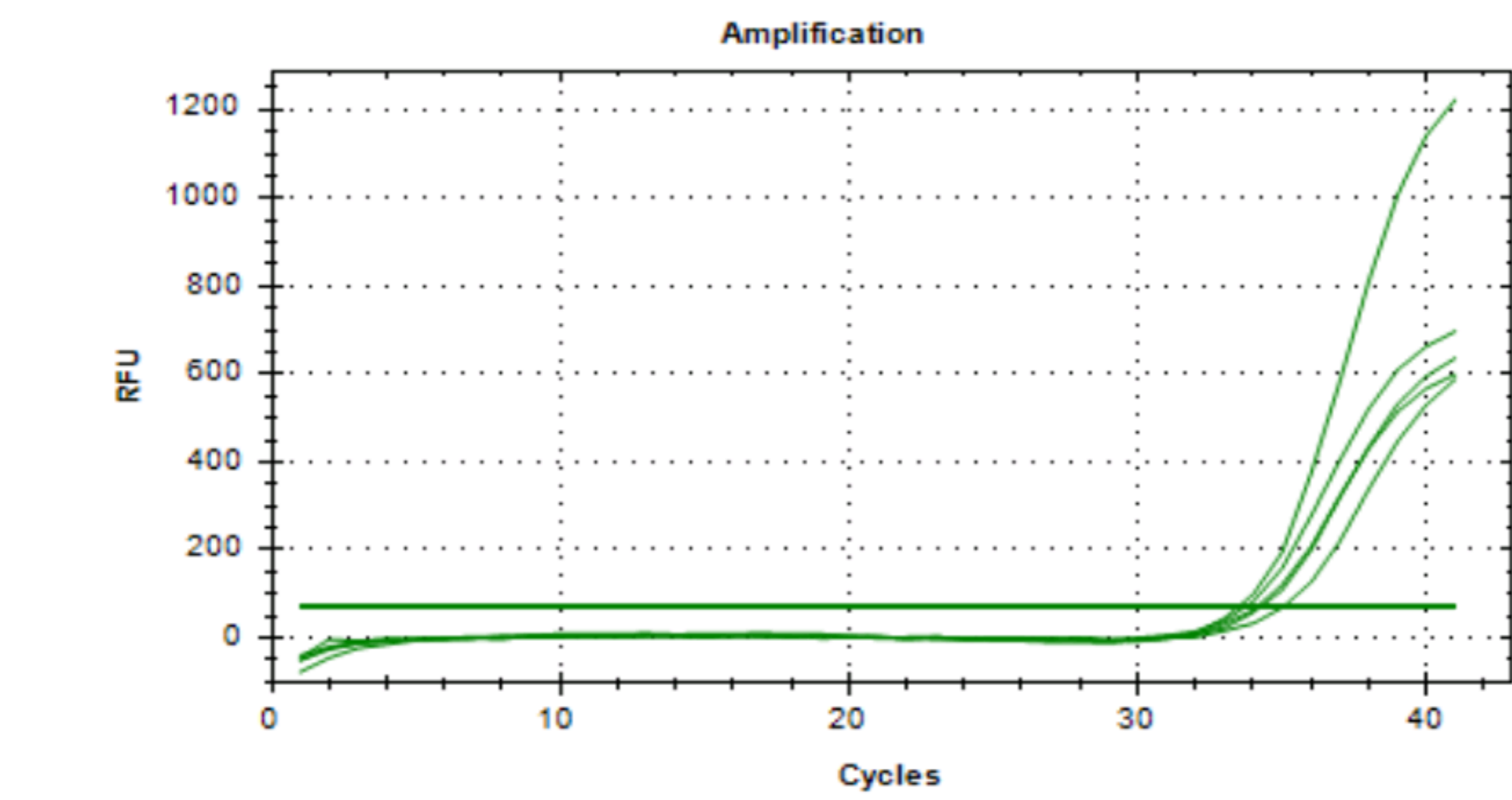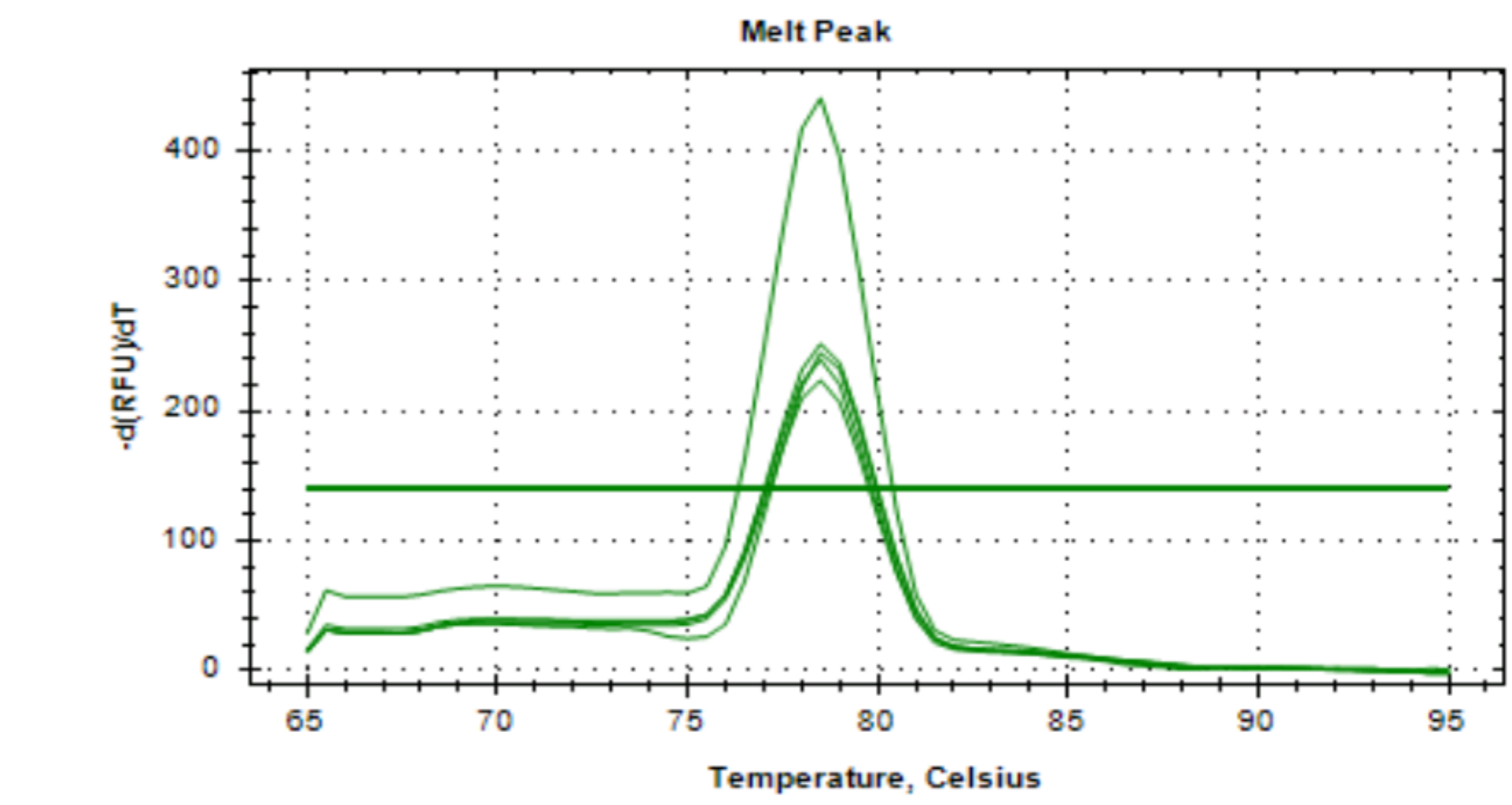

GAPDH

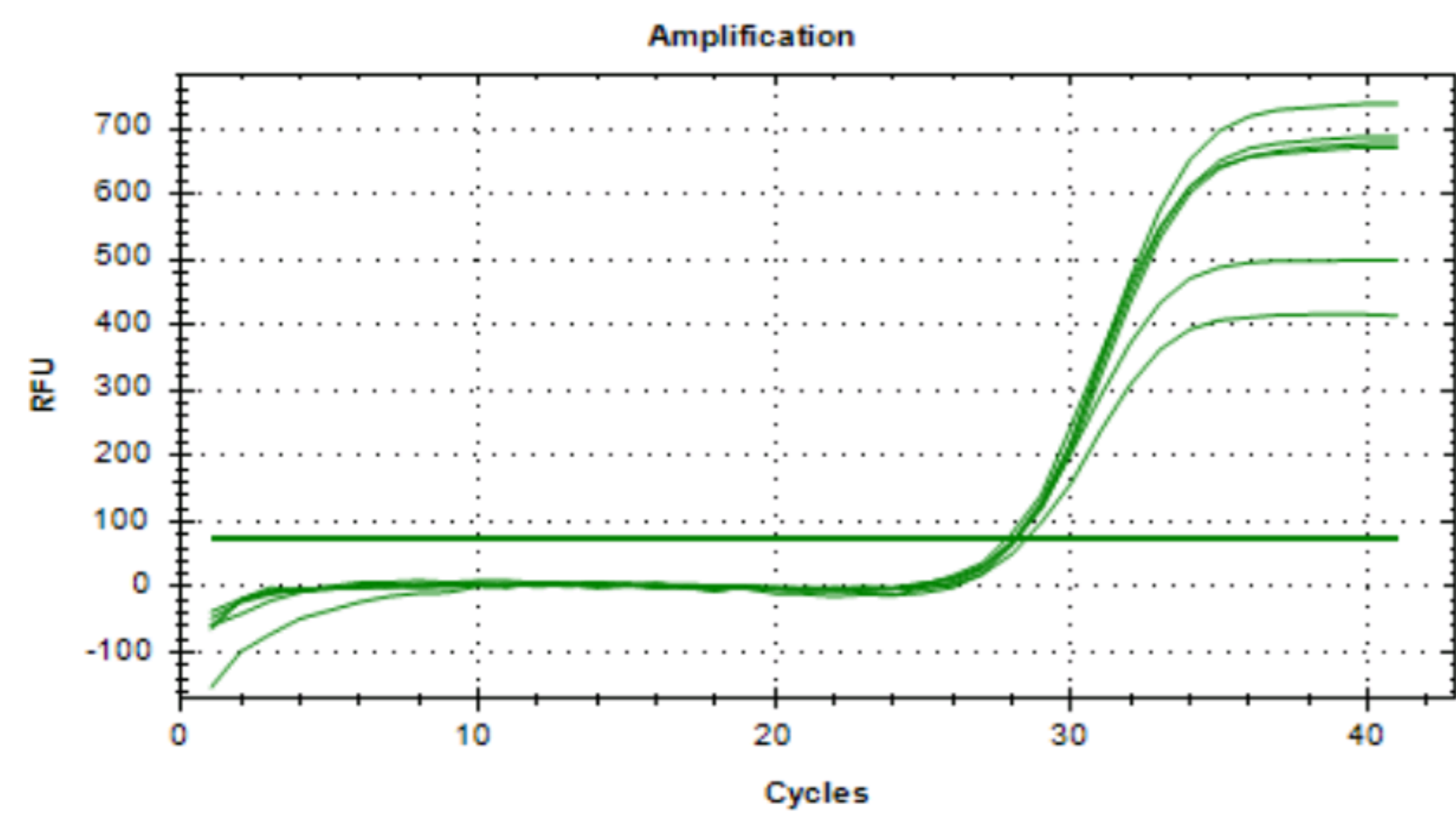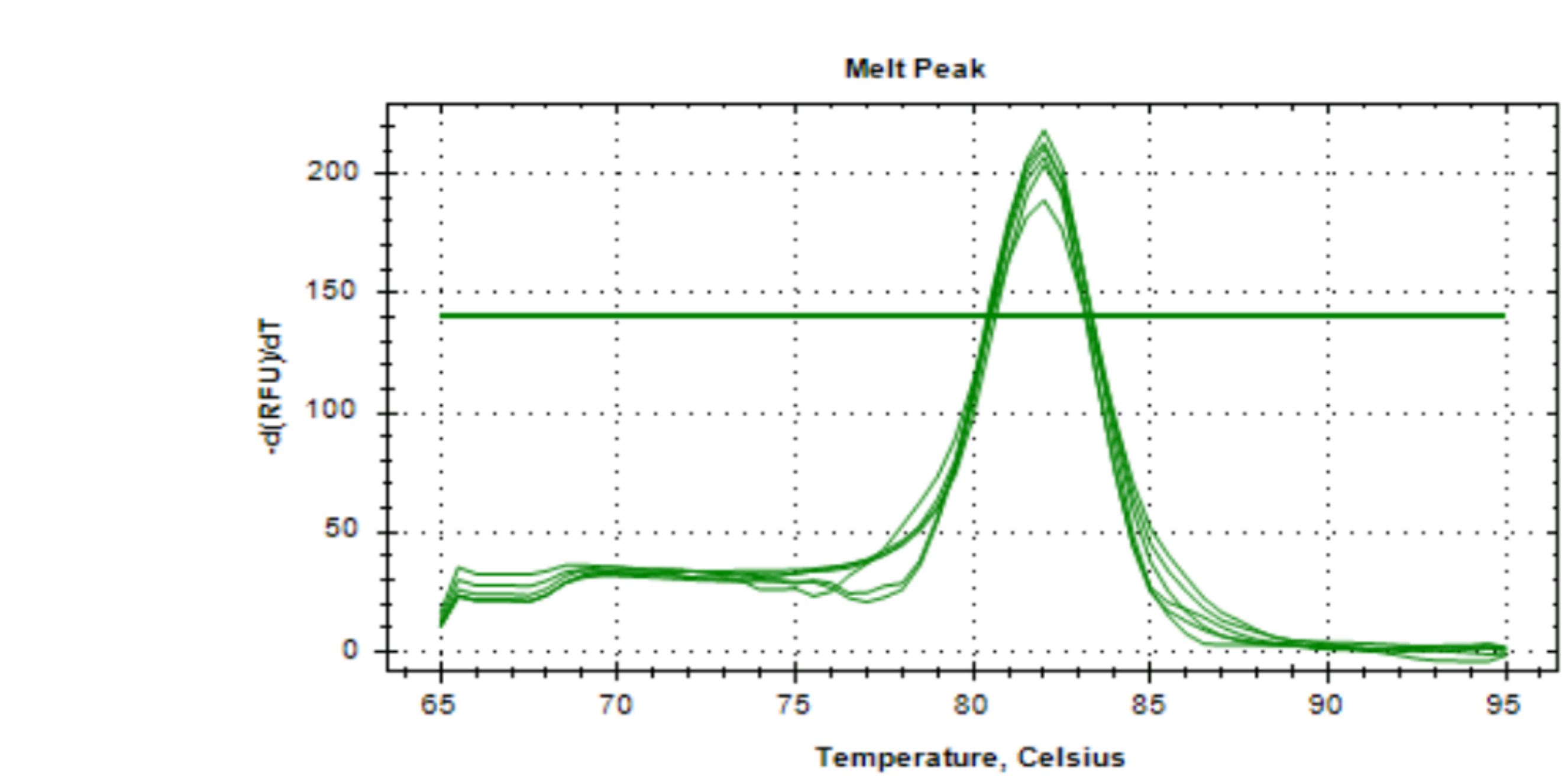

Supplement: Supplementary file 3 — Supplementary Information 3. [file 41598_2024_57122_MOESM3_ESM.pdf]

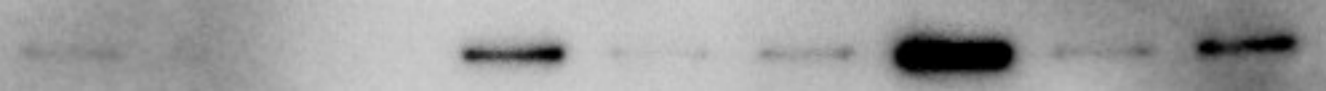

Supplement: Supplementary file 4 — Supplementary Information 4. [file 41598_2024_57122_MOESM4_ESM.pdf]

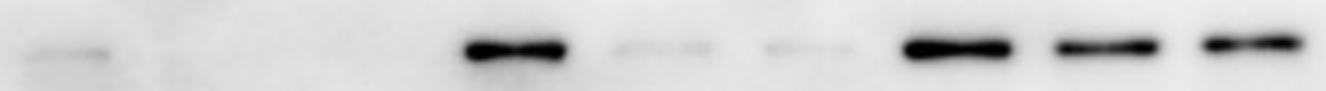

Supplement: Supplementary file 5 — Supplementary Information 5. [file 41598_2024_57122_MOESM5_ESM.pdf]
