# Supplementary material for: Mechanistic study on the alleviation of postmenopausal osteoporosis by Lactobacillus acidophilus through butyrate-mediated inhibition of osteoclast activity
Source: Sci Rep. 2024 Mar 25;14:7042. doi: 10.1038/s41598-024-57122-x (PMC10963762; doi:10.1038/s41598-024-57122-x)
Supplement: Supplementary file 6 — Supplementary Information 6. [file 41598_2024_57122_MOESM6_ESM.pptx]

## Slide 1
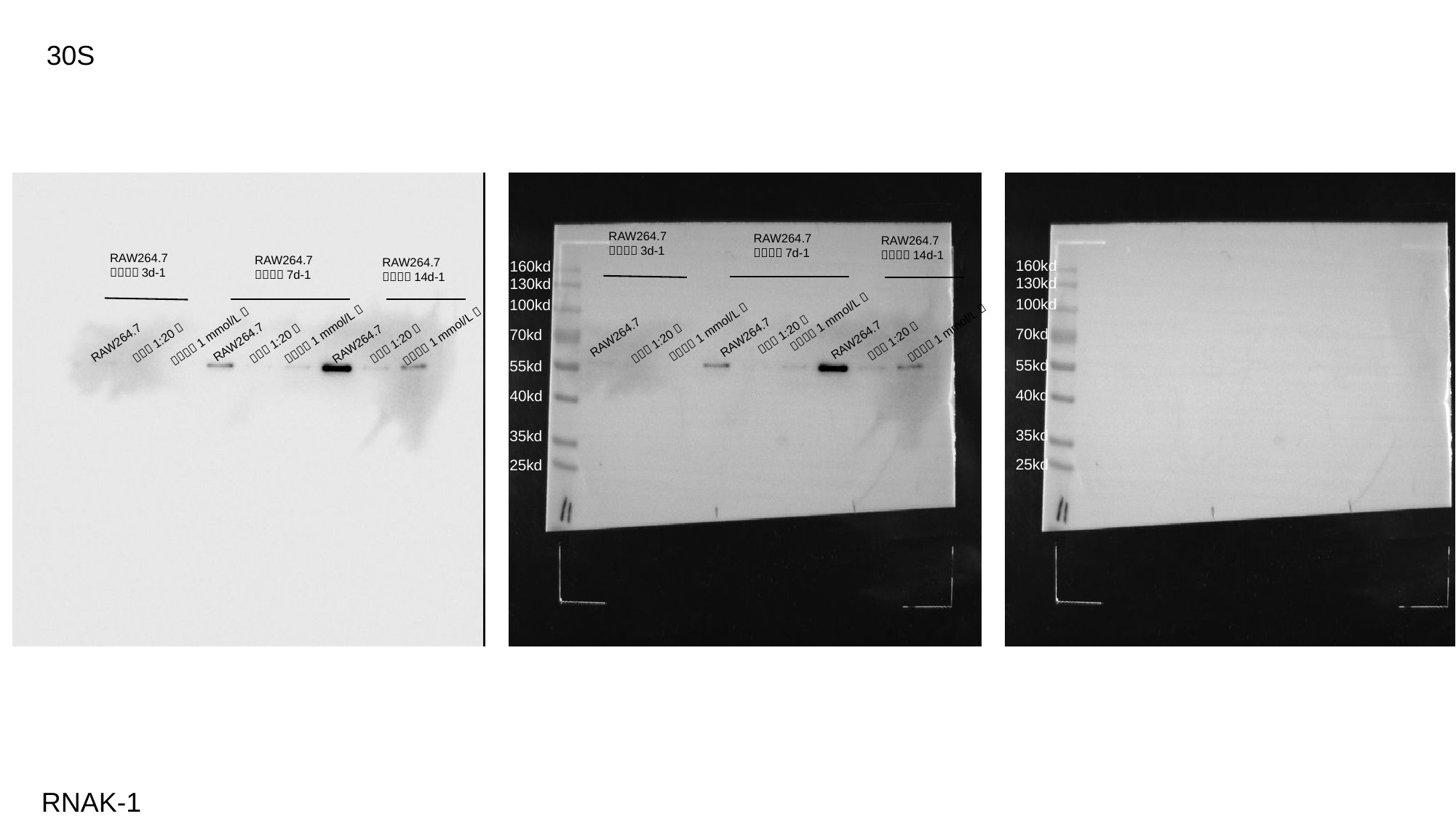

30S
RAW264.7
破骨诱导3d-1
RAW264.7
破骨诱导7d-1
RAW264.7
破骨诱导14d-1
RAW264.7
破骨诱导3d-1
RAW264.7
破骨诱导7d-1
RAW264.7
破骨诱导14d-1
160kd
160kd
丁酸钠（1 mmol/L）
130kd
130kd
菌液（1:20）
RAW264.7
RAW264.7
RAW264.7
丁酸钠（1 mmol/L）
RAW264.7
RAW264.7
菌液（1:20）
菌液（1:20）
菌液（1:20）
丁酸钠（1 mmol/L）
RAW264.7
丁酸钠（1 mmol/L）
菌液（1:20）
100kd
100kd
菌液（1:20）
丁酸钠（1 mmol/L）
丁酸钠（1 mmol/L）
70kd
70kd
55kd
55kd
40kd
40kd
35kd
35kd
25kd
25kd
RNAK-1

## Slide 2
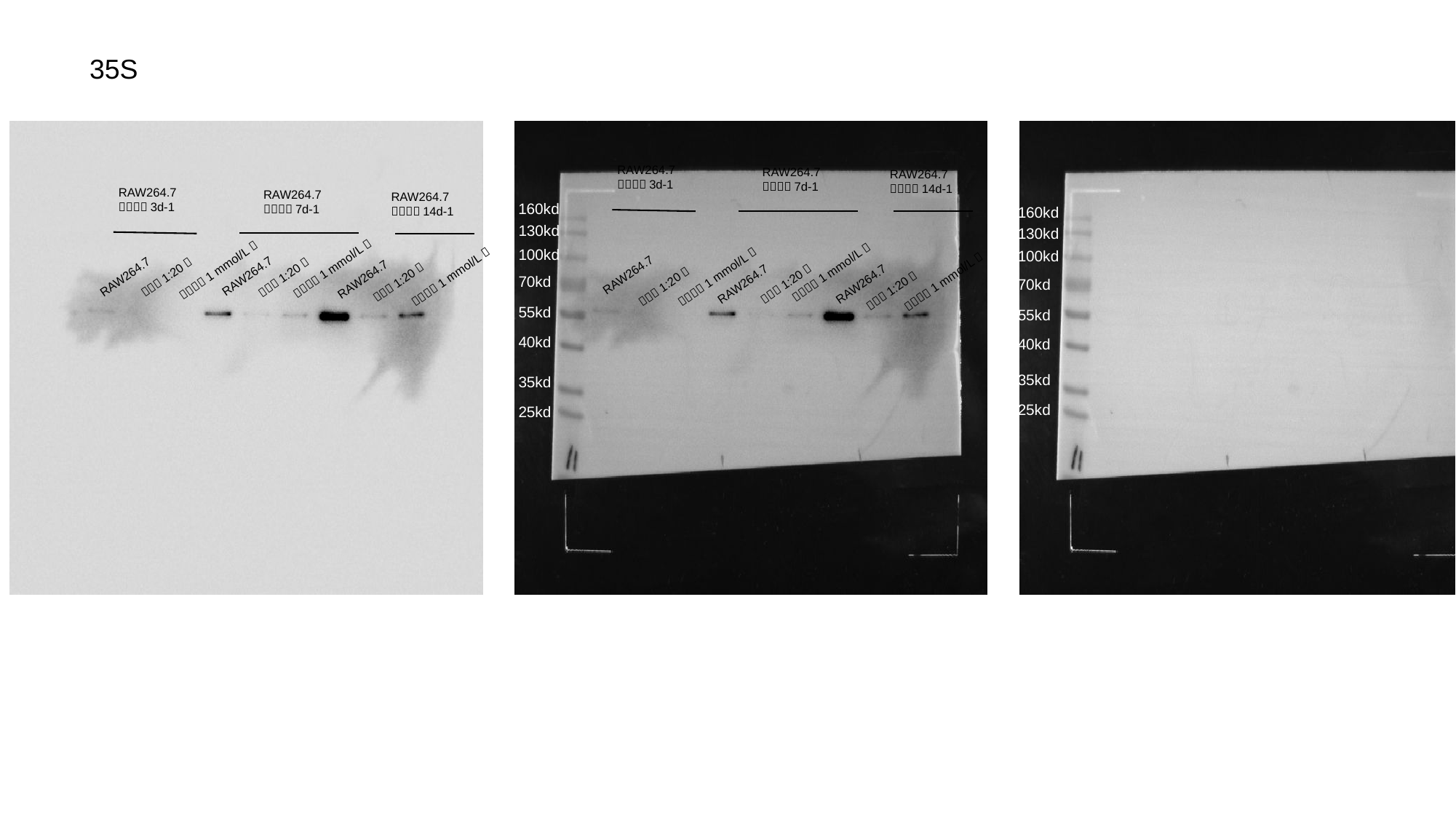

35S
RAW264.7
破骨诱导3d-1
RAW264.7
破骨诱导7d-1
RAW264.7
破骨诱导14d-1
RAW264.7
破骨诱导3d-1
RAW264.7
破骨诱导7d-1
RAW264.7
破骨诱导14d-1
160kd
160kd
RAW264.7
RAW264.7
RAW264.7
菌液（1:20）
菌液（1:20）
丁酸钠（1 mmol/L）
RAW264.7
丁酸钠（1 mmol/L）
130kd
丁酸钠（1 mmol/L）
130kd
菌液（1:20）
RAW264.7
RAW264.7
菌液（1:20）
丁酸钠（1 mmol/L）
丁酸钠（1 mmol/L）
丁酸钠（1 mmol/L）
菌液（1:20）
菌液（1:20）
100kd
100kd
70kd
70kd
55kd
55kd
40kd
40kd
35kd
35kd
25kd
25kd

## Slide 3
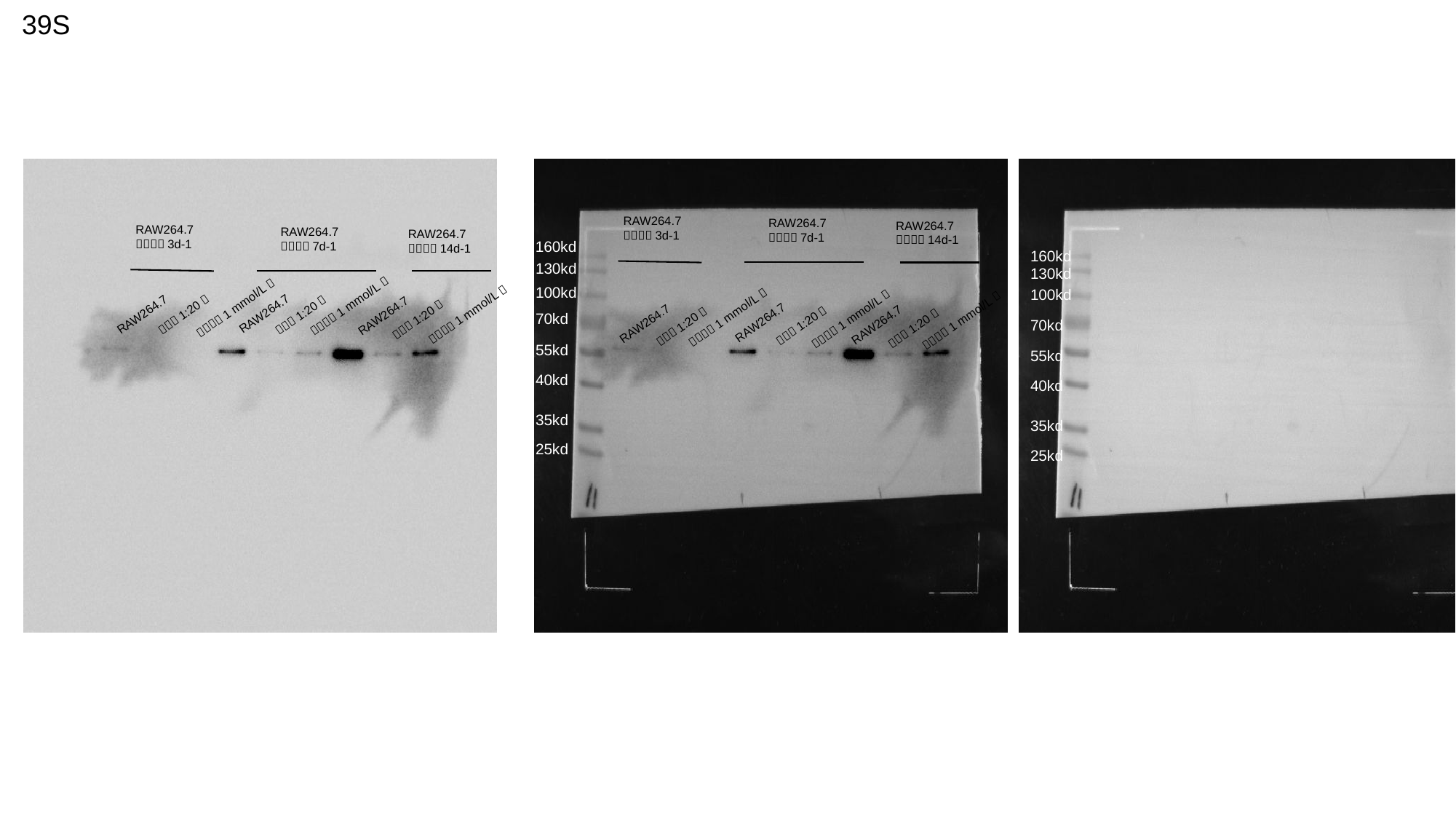

39S
RAW264.7
破骨诱导3d-1
RAW264.7
破骨诱导7d-1
RAW264.7
破骨诱导14d-1
RAW264.7
破骨诱导3d-1
RAW264.7
破骨诱导7d-1
RAW264.7
破骨诱导14d-1
160kd
160kd
RAW264.7
RAW264.7
菌液（1:20）
菌液（1:20）
丁酸钠（1 mmol/L）
RAW264.7
丁酸钠（1 mmol/L）
130kd
RAW264.7
130kd
RAW264.7
丁酸钠（1 mmol/L）
RAW264.7
菌液（1:20）
菌液（1:20）
丁酸钠（1 mmol/L）
丁酸钠（1 mmol/L）
菌液（1:20）
菌液（1:20）
100kd
100kd
丁酸钠（1 mmol/L）
70kd
70kd
55kd
55kd
40kd
40kd
35kd
35kd
25kd
25kd

## Slide 4
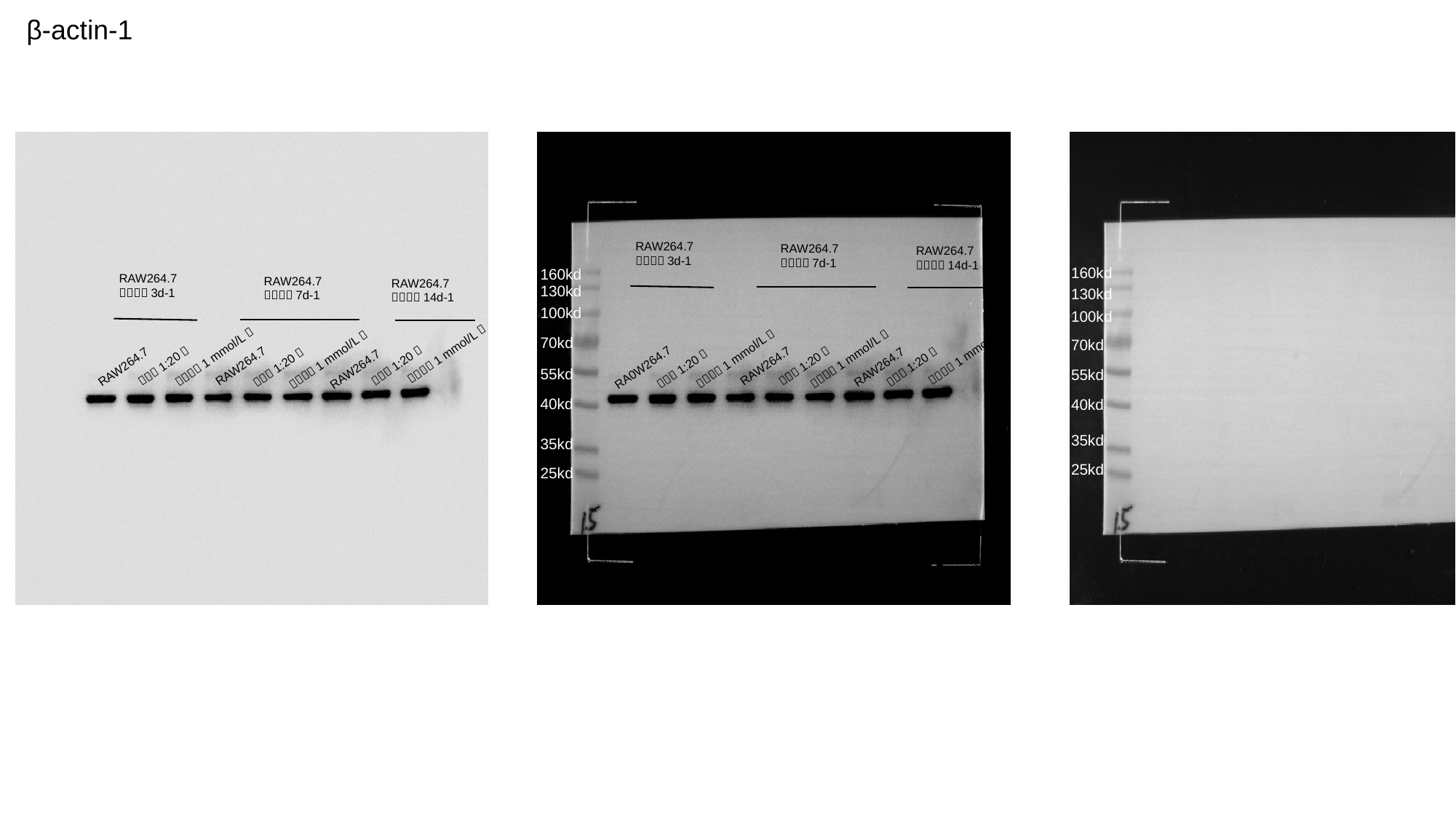

β-actin-1
RAW264.7
破骨诱导3d-1
RAW264.7
破骨诱导7d-1
RAW264.7
破骨诱导14d-1
160kd
160kd
RAW264.7
破骨诱导3d-1
RAW264.7
破骨诱导7d-1
RAW264.7
破骨诱导14d-1
130kd
130kd
100kd
丁酸钠（1 mmol/L）
100kd
菌液（1:20）
菌液（1:20）
RAW264.7
RAW264.7
丁酸钠（1 mmol/L）
RAW264.7
RAW264.7
菌液（1:20）
丁酸钠（1 mmol/L）
丁酸钠（1 mmol/L）
丁酸钠（1 mmol/L）
菌液（1:20）
RAW264.7
RA0W264.7
菌液（1:20）
菌液（1:20）
丁酸钠（1 mmol/L）
70kd
70kd
55kd
55kd
40kd
40kd
35kd
35kd
25kd
25kd

## Slide 5
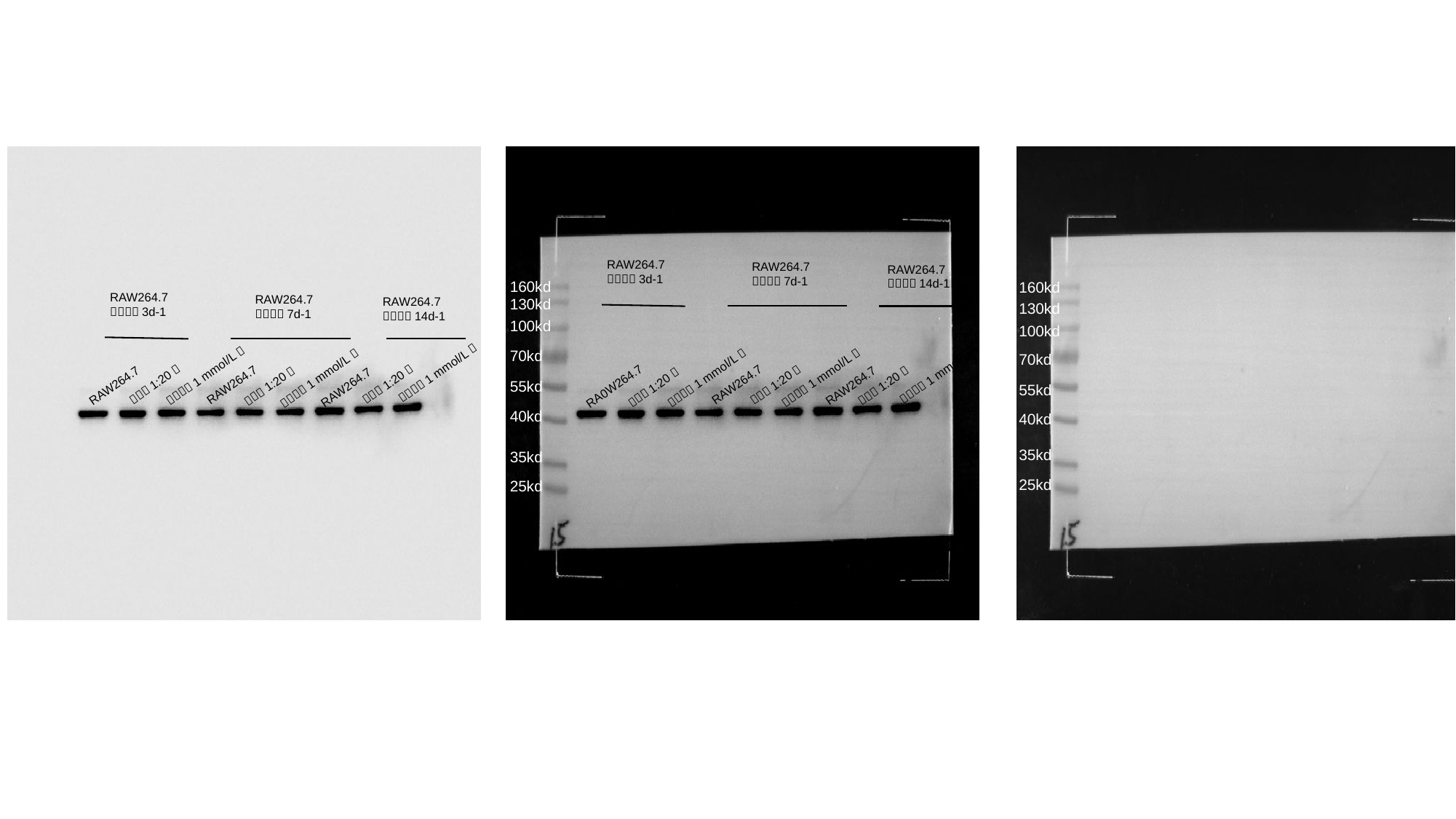

RAW264.7
破骨诱导3d-1
RAW264.7
破骨诱导7d-1
RAW264.7
破骨诱导14d-1
160kd
160kd
RAW264.7
破骨诱导3d-1
RAW264.7
破骨诱导7d-1
RAW264.7
破骨诱导14d-1
130kd
130kd
100kd
100kd
丁酸钠（1 mmol/L）
菌液（1:20）
菌液（1:20）
RAW264.7
RAW264.7
丁酸钠（1 mmol/L）
RAW264.7
RAW264.7
菌液（1:20）
丁酸钠（1 mmol/L）
丁酸钠（1 mmol/L）
丁酸钠（1 mmol/L）
菌液（1:20）
RA0W264.7
菌液（1:20）
RAW264.7
70kd
菌液（1:20）
70kd
丁酸钠（1 mmol/L）
55kd
55kd
40kd
40kd
35kd
35kd
25kd
25kd

## Slide 6
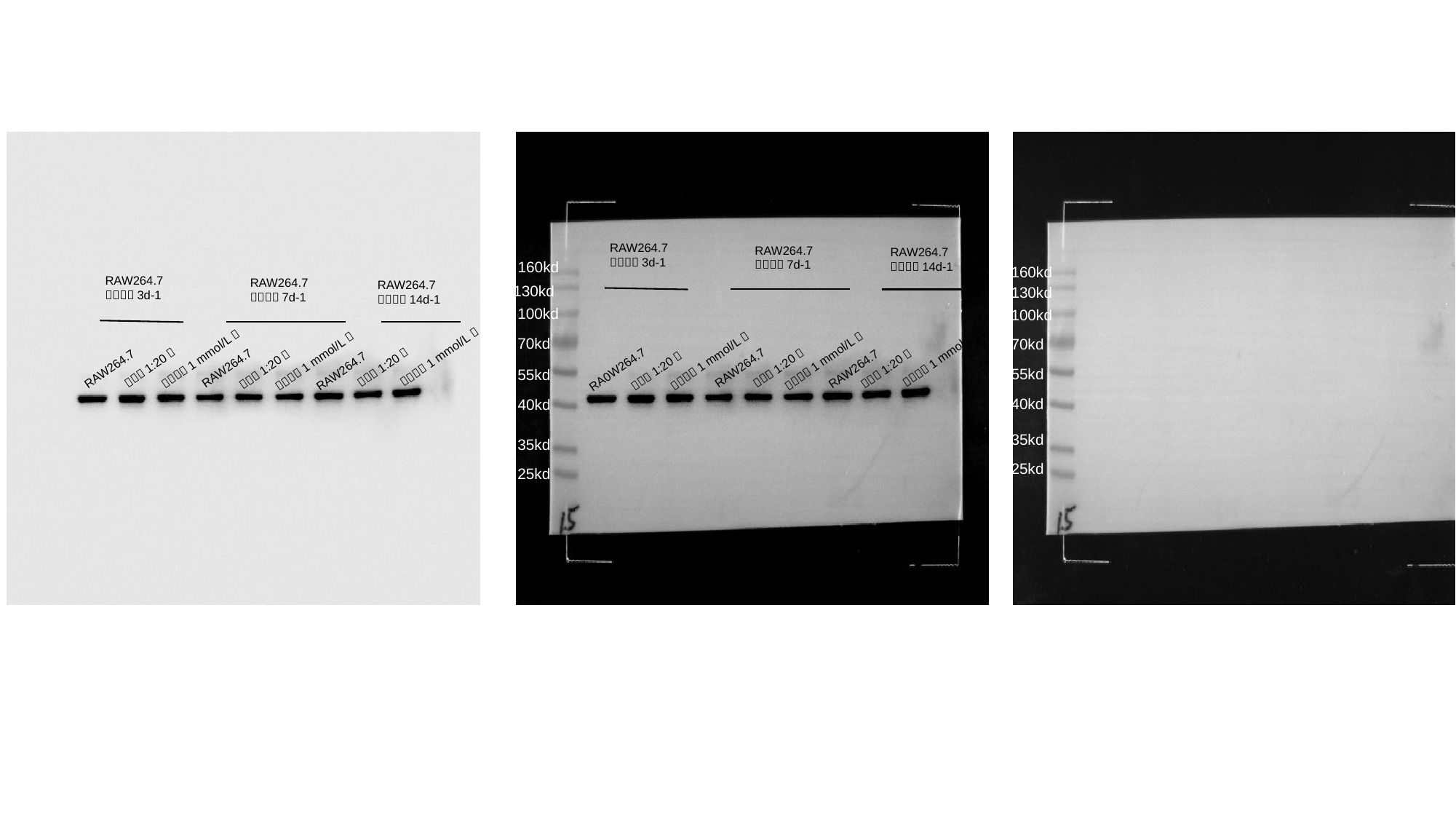

RAW264.7
破骨诱导3d-1
RAW264.7
破骨诱导7d-1
RAW264.7
破骨诱导14d-1
160kd
160kd
RAW264.7
破骨诱导3d-1
RAW264.7
破骨诱导7d-1
RAW264.7
破骨诱导14d-1
130kd
130kd
100kd
100kd
丁酸钠（1 mmol/L）
菌液（1:20）
菌液（1:20）
RAW264.7
RAW264.7
丁酸钠（1 mmol/L）
RAW264.7
RAW264.7
菌液（1:20）
丁酸钠（1 mmol/L）
丁酸钠（1 mmol/L）
丁酸钠（1 mmol/L）
菌液（1:20）
RAW264.7
RA0W264.7
菌液（1:20）
菌液（1:20）
70kd
70kd
丁酸钠（1 mmol/L）
55kd
55kd
40kd
40kd
35kd
35kd
25kd
25kd

## Slide 7
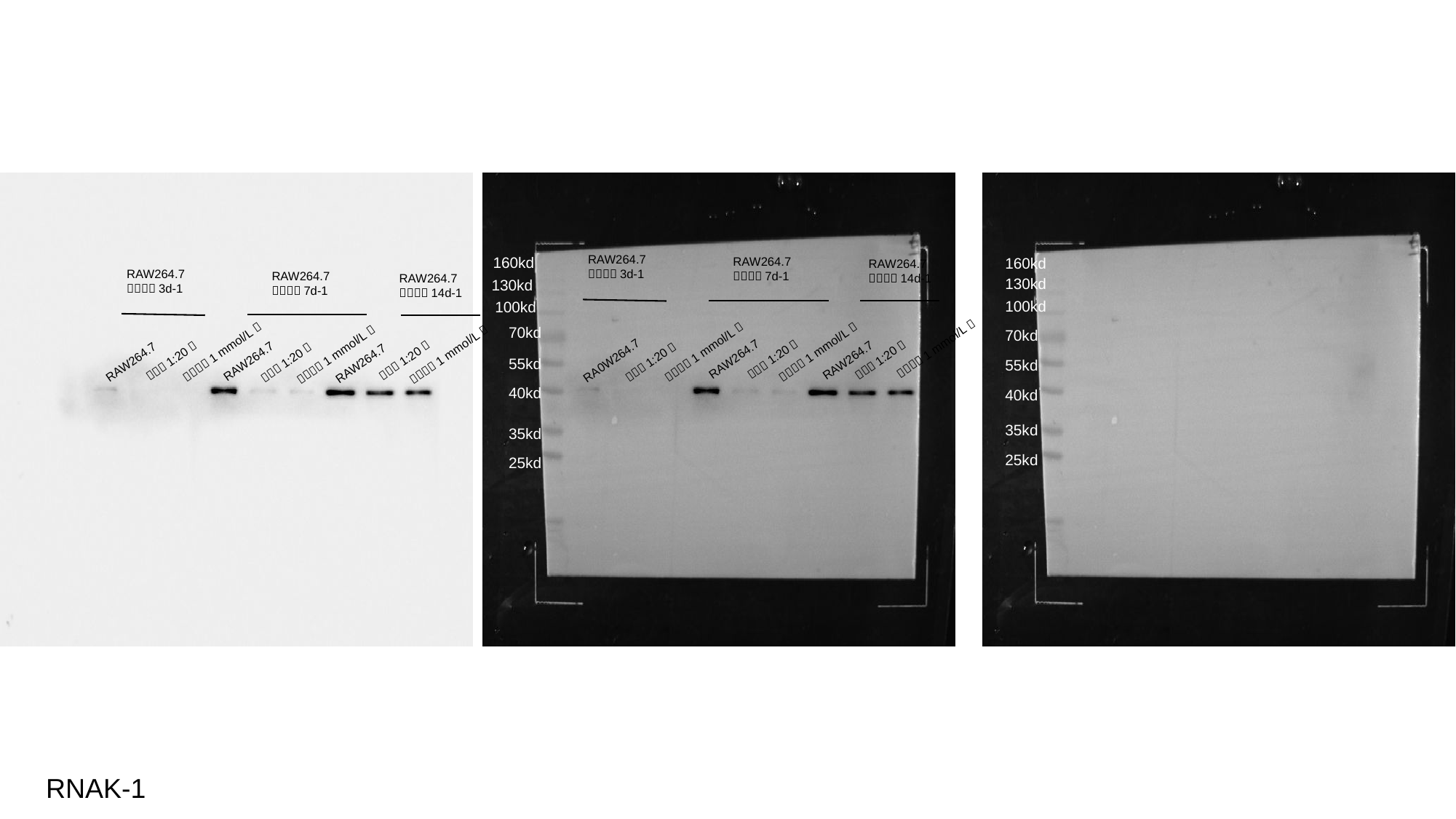

RAW264.7
破骨诱导3d-1
160kd
160kd
RAW264.7
破骨诱导7d-1
RAW264.7
破骨诱导14d-1
RAW264.7
破骨诱导3d-1
RAW264.7
破骨诱导7d-1
RAW264.7
破骨诱导14d-1
130kd
130kd
100kd
100kd
丁酸钠（1 mmol/L）
菌液（1:20）
RAW264.7
RAW264.7
菌液（1:20）
丁酸钠（1 mmol/L）
RAW264.7
丁酸钠（1 mmol/L）
丁酸钠（1 mmol/L）
菌液（1:20）
RAW264.7
菌液（1:20）
RA0W264.7
丁酸钠（1 mmol/L）
菌液（1:20）
RAW264.7
70kd
70kd
菌液（1:20）
丁酸钠（1 mmol/L）
55kd
55kd
40kd
40kd
35kd
35kd
25kd
25kd
RNAK-1

## Slide 8
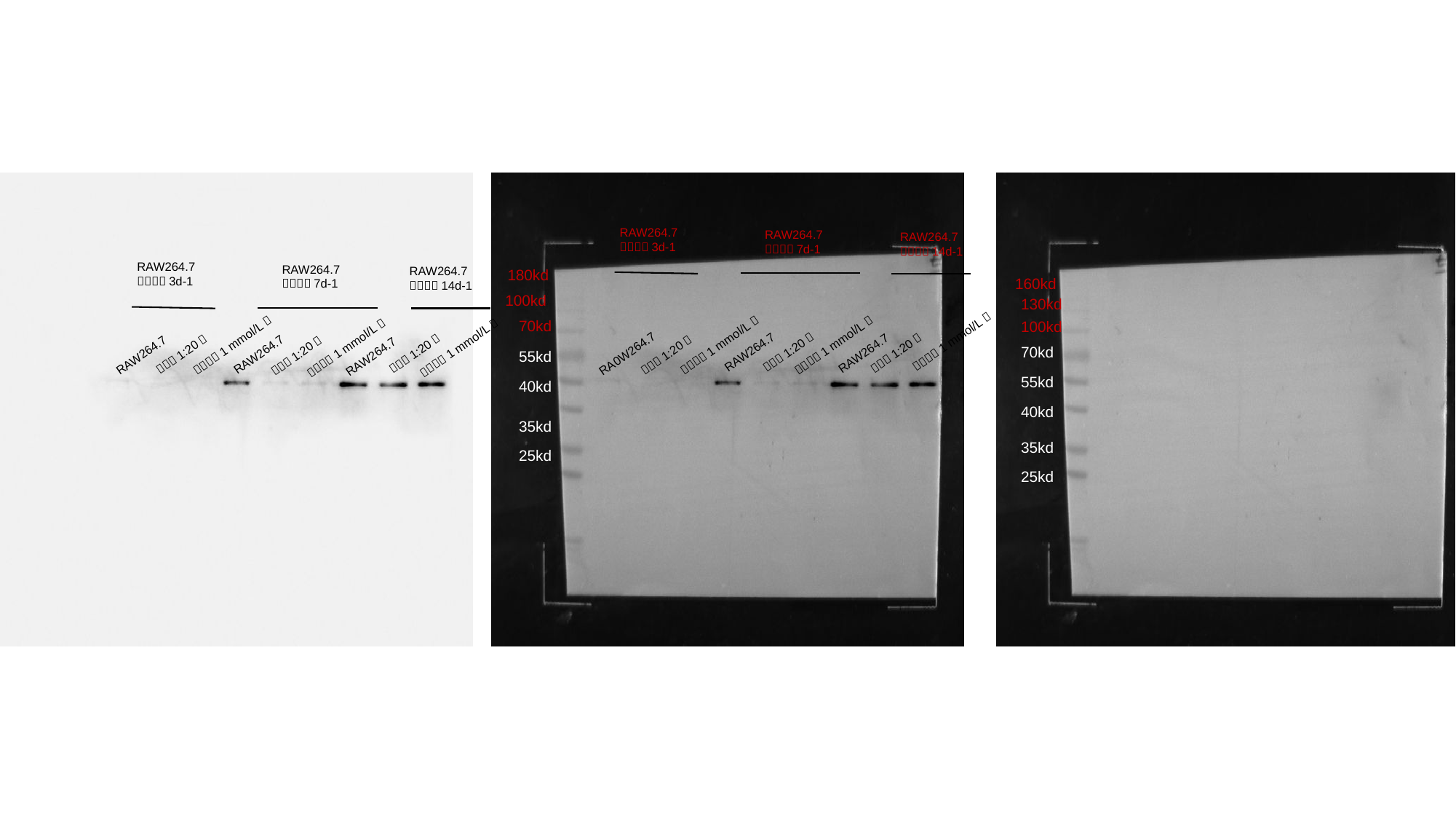

RAW264.7
破骨诱导3d-1
RAW264.7
破骨诱导7d-1
RAW264.7
破骨诱导14d-1
RAW264.7
破骨诱导3d-1
RAW264.7
破骨诱导7d-1
RAW264.7
破骨诱导14d-1
180kd
160kd
100kd
丁酸钠（1 mmol/L）
菌液（1:20）
RAW264.7
130kd
RAW264.7
菌液（1:20）
丁酸钠（1 mmol/L）
RAW264.7
丁酸钠（1 mmol/L）
丁酸钠（1 mmol/L）
菌液（1:20）
RAW264.7
菌液（1:20）
RA0W264.7
丁酸钠（1 mmol/L）
菌液（1:20）
RAW264.7
70kd
100kd
菌液（1:20）
丁酸钠（1 mmol/L）
70kd
55kd
55kd
40kd
40kd
35kd
35kd
25kd
25kd

## Slide 9
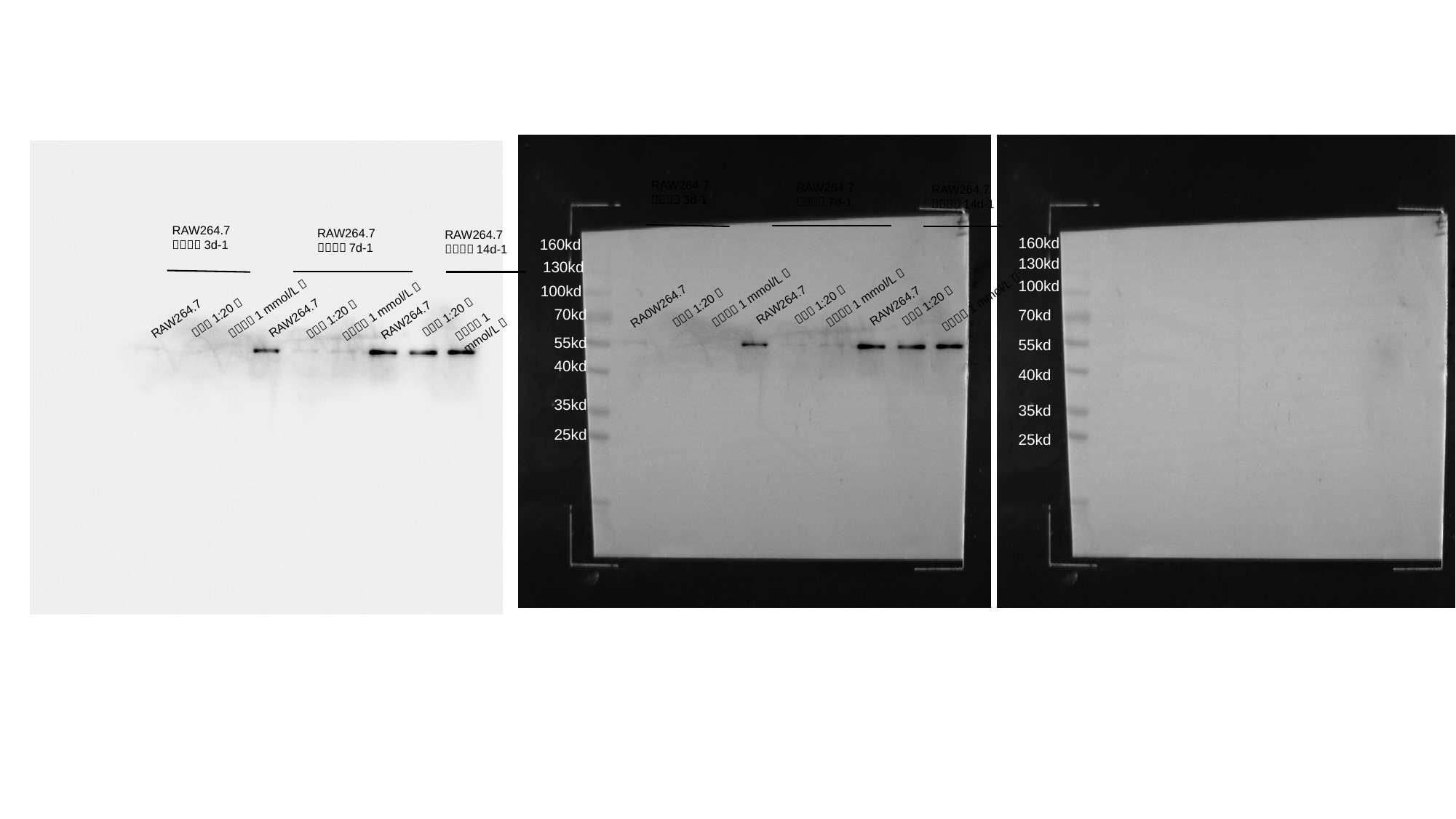

RAW264.7
破骨诱导3d-1
RAW264.7
破骨诱导7d-1
RAW264.7
破骨诱导14d-1
RAW264.7
破骨诱导3d-1
RAW264.7
破骨诱导7d-1
RAW264.7
破骨诱导14d-1
160kd
160kd
菌液（1:20）
RAW264.7
RAW264.7
丁酸钠（1 mmol/L）
丁酸钠（1 mmol/L）
菌液（1:20）
RA0W264.7
丁酸钠（1 mmol/L）
130kd
菌液（1:20）
130kd
菌液（1:20）
RAW264.7
丁酸钠（1 mmol/L）
RAW264.7
菌液（1:20）
丁酸钠（1 mmol/L）
100kd
RAW264.7
100kd
菌液（1:20）
70kd
70kd
丁酸钠（1 mmol/L）
55kd
55kd
40kd
40kd
35kd
35kd
25kd
25kd

## Slide 10
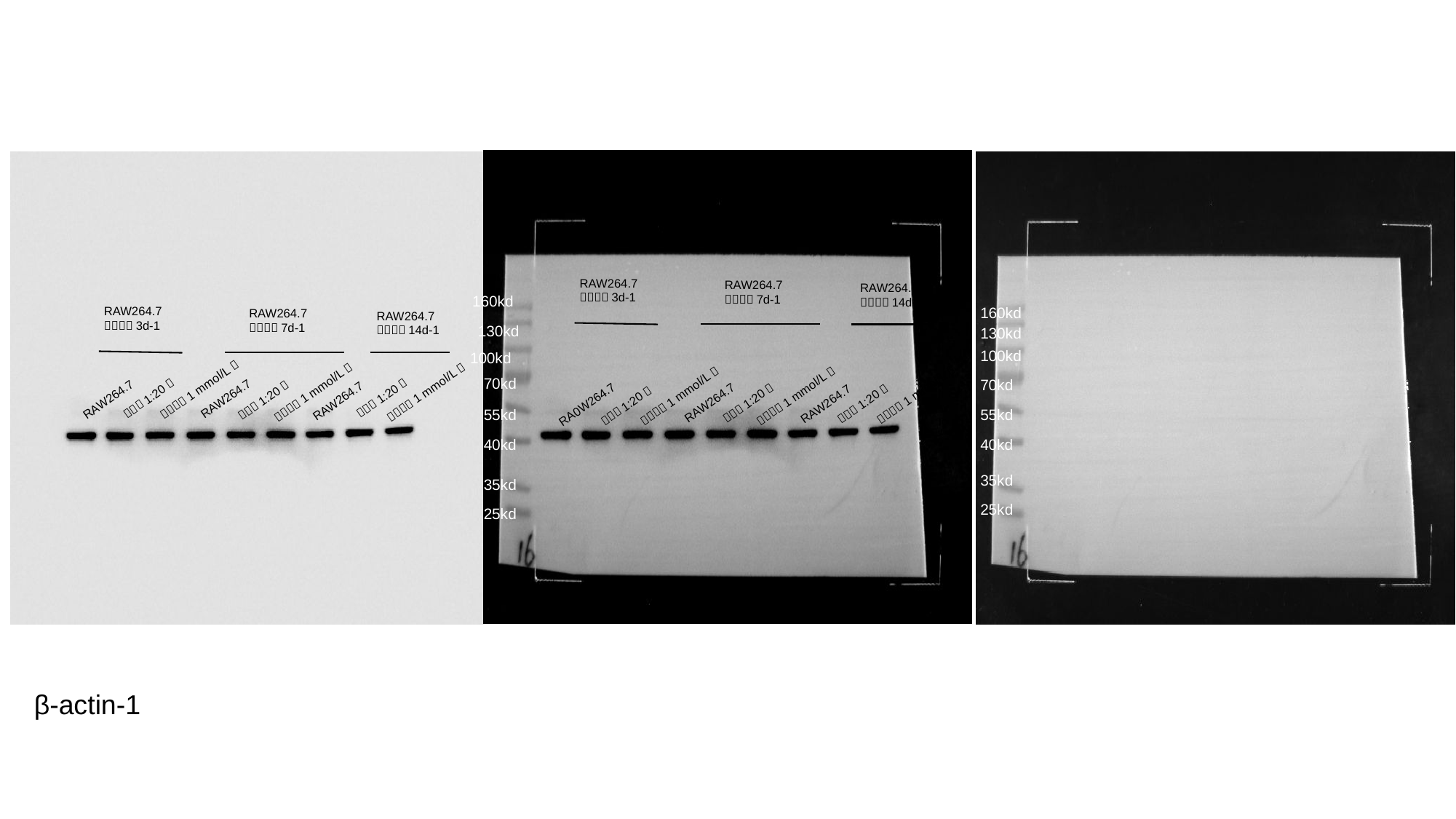

RAW264.7
破骨诱导3d-1
RAW264.7
破骨诱导7d-1
RAW264.7
破骨诱导14d-1
160kd
160kd
RAW264.7
破骨诱导3d-1
RAW264.7
破骨诱导7d-1
RAW264.7
破骨诱导14d-1
130kd
130kd
菌液（1:20）
RAW264.7
丁酸钠（1 mmol/L）
RAW264.7
菌液（1:20）
丁酸钠（1 mmol/L）
菌液（1:20）
RAW264.7
RAW264.7
丁酸钠（1 mmol/L）
丁酸钠（1 mmol/L）
100kd
丁酸钠（1 mmol/L）
菌液（1:20）
RA0W264.7
100kd
菌液（1:20）
RAW264.7
菌液（1:20）
70kd
70kd
丁酸钠（1 mmol/L）
55kd
55kd
40kd
40kd
35kd
35kd
25kd
25kd
β-actin-1

## Slide 11
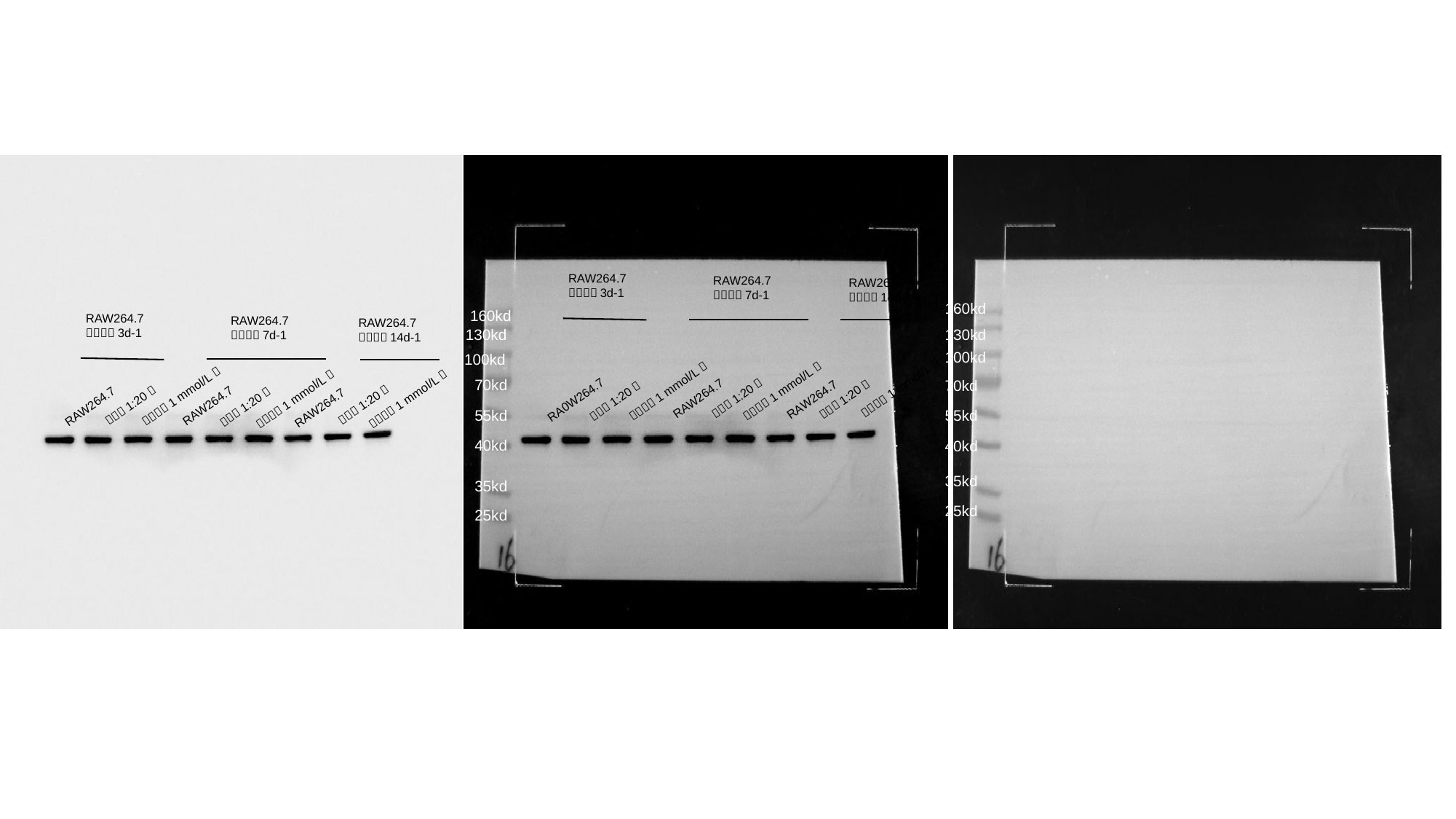

RAW264.7
破骨诱导3d-1
RAW264.7
破骨诱导7d-1
RAW264.7
破骨诱导14d-1
160kd
160kd
RAW264.7
破骨诱导3d-1
RAW264.7
破骨诱导7d-1
RAW264.7
破骨诱导14d-1
130kd
130kd
丁酸钠（1 mmol/L）
菌液（1:20）
RAW264.7
RAW264.7
丁酸钠（1 mmol/L）
丁酸钠（1 mmol/L）
菌液（1:20）
RA0W264.7
菌液（1:20）
RAW264.7
丁酸钠（1 mmol/L）
RAW264.7
100kd
菌液（1:20）
丁酸钠（1 mmol/L）
100kd
菌液（1:20）
RAW264.7
菌液（1:20）
70kd
70kd
丁酸钠（1 mmol/L）
55kd
55kd
40kd
40kd
35kd
35kd
25kd
25kd

## Slide 12
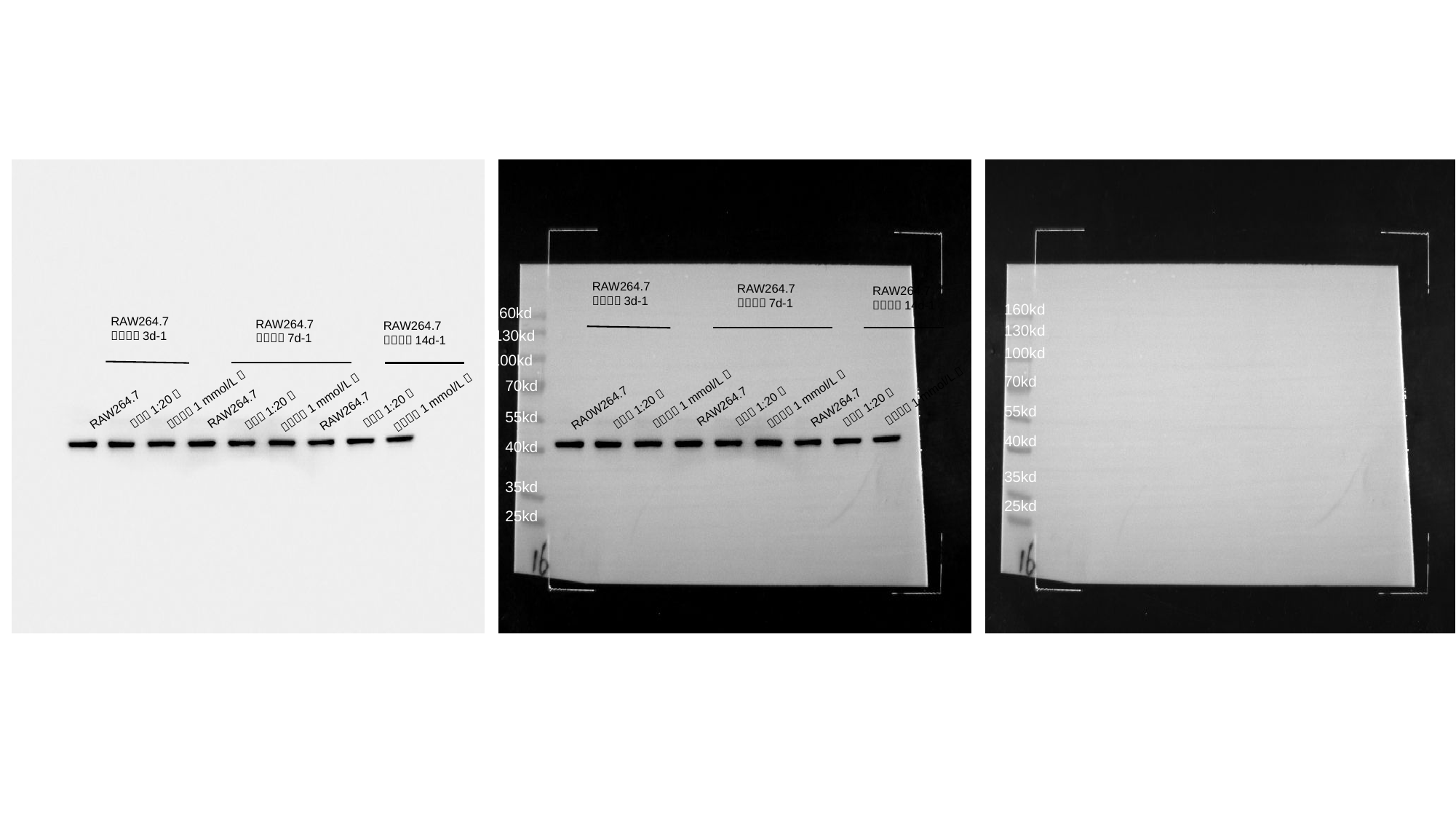

RAW264.7
破骨诱导3d-1
RAW264.7
破骨诱导7d-1
RAW264.7
破骨诱导14d-1
160kd
160kd
RAW264.7
破骨诱导3d-1
RAW264.7
破骨诱导7d-1
RAW264.7
破骨诱导14d-1
130kd
130kd
100kd
丁酸钠（1 mmol/L）
菌液（1:20）
RAW264.7
RAW264.7
丁酸钠（1 mmol/L）
菌液（1:20）
丁酸钠（1 mmol/L）
菌液（1:20）
RAW264.7
丁酸钠（1 mmol/L）
RAW264.7
100kd
RA0W264.7
菌液（1:20）
丁酸钠（1 mmol/L）
菌液（1:20）
RAW264.7
70kd
菌液（1:20）
70kd
丁酸钠（1 mmol/L）
55kd
55kd
40kd
40kd
35kd
35kd
25kd
25kd
